# Impaired cardiac non-neuronal acetylcholine synthesis triggers mitochondrial dysfunction with the loss of nicotinic receptor-mediated calcium handling, causing the failing heart

**DOI:** 10.1042/CS20257026

**Published:** 2025-11-25

**Authors:** Takashi Sonobe, Yuko Kai, Shino Oikawa, Takumi Akagi, Asuka Mano, Rimpei Morita, Masayuki Tsuda, Yoshihiko Kakinuma

**Affiliations:** 1 Department of Bioregulatory Science, Nippon Medical School, 113-8602, Japan; 2 Department of Physiology, Nippon Medical School, 113-8602, Japan; 3 Department of Microbiology and Immunology, Nippon Medical School, 113-8602, Japan; 4 Institute for Laboratory Animal Research, Kochi Medical School, Nankoku, Kochi, 783-8505, Japan

**Keywords:** α7 nicotinic ACh receptor (nAChR), acetylcholine (ACh), autophagy, blood–brain barrier (BBB), cardiac dysfunction, cardiac energy metabolism, choline acetyltransferase (ChAT), higher brain function, inflammation, mitochondria, mitochondrial calcium uniporter (MCU), non-neuronal cardiac cholinergic system (NNCCS)

## Abstract

Our previous studies, as well as other investigations, demonstrated that non-neuronal acetylcholine (ACh) produced by cardiomyocytes—that is, the non-neuronal cardiac cholinergic system (NNCCS)—is indispensable for sustaining the physiological functions and structural integrity of cardiomyocytes and for protecting the heart from ischemic/hypoxic insults, hypertrophic stress, and hypersympathetic conditions. These findings were supported by pharmacologically manipulated models in non-neuronal ACh systems and by gain- or loss-of-function models in the NNCCS. Nevertheless, the mechanisms underlying this phenomenon (i.e., sustention and protection) and the target of the NNCCS in cardiomyocytes remain to be fully elucidated. Our conditional murine model with heart-specific deletion of the choline acetyltransferase (ChAT) gene in the heart (hChAT KO mice) revealed cardiac dysfunction associated with heart failure symptoms. The representative culprit targets were the mitochondria with a disorganized appearance and dysfunction, accompanied by a reduction in mitochondrial DNA, membrane potential, and ATP production. Alternatively, malfunctioning mitochondria impaired cardiac energy metabolism and nicotinic receptor-mediated calcium responses in the mitochondria and down-regulated the mitochondrial calcium uniporter (MCU), leading to poor calcium handling by the mitochondria. The impaired cardiac function in hChAT KO mice induced systemic inflammatory responses and attenuated blood–brain barrier function, further influencing higher brain functions, including the aggravation of depression-like phenomenon. These specifically characteristic phenotypes indicate that the NNCCS principally plays a crucial role in sustaining mitochondrial functions through nicotinic receptors in the mitochondria and that the signal is indispensable for maintaining mitochondrial functions and integrity.

## Introduction

Non-neuronal acetylcholine (ACh) is produced by cells other than neuronal cells because they are equipped with the necessary components to synthesize and store ACh, including a choline transporter, the ACh synthesis enzyme choline acetyltransferase (ChAT), and the vesicular ACh transporter VAChT [1]. Previous studies have reported that immune cells such as macrophages, T cells, bronchial epithelial cells, and endothelial cells produce ACh [2–4], which plays specific physiological roles in adaption to a particular cellular environment, independent of the parasympathetic nervous system (PNS) [2,4,5]. Similarly, several important studies on the cardiovascular system have provided sufficient evidence that cardiomyocytes synthesize ACh independent of the PNS, which mainly innervates the sinus and atrioventricular nodes in the conduction system of the heart; however, innervates the ventricles less than the atria [6–10]. Thus, the source of cardiomyocyte-derived ACh differs from that of ACh from the terminals of the PNS with respect to anatomical location. Since this report [10], this system has been called ‘non-neuronal ACh’ in cardiomyocytes/hearts or the non-neuronal cardiac cholinergic system (NNCCS) [11]. Several crucial studies conducted globally have suggested that the NNCCS plays a pivotal role in the heart, both physiologically and pathophysiologically [6–8, 10]. Physiologically, the NNCCS is essential for negatively regulating oxygen consumption, maintaining gap junction functions, accelerating angiogenesis via cardiomyocyte-derived ACh, and protecting the heart from exposure to norepinephrine, which is released from the terminals of the sympathetic nervous system innervating the entire heart [10,12,13]. Therefore, it plays a protective role in the heart against hypertrophic, ischemic, and hypoxic insults in pathological conditions [7, 8, 13–14]. These striking findings were obtained using genetically modified mice [8,13,14] and cells [12] or utilizing pharmacological approaches [7,10,12]. Despite these findings, the specific downstream molecules or targets of the NNCCS and their precise mechanisms are not fully understood. Moreover, the precise link between the NNCCS function and intracellular organelles in cardiomyocytes remains unclear [14].

To address the aforementioned issues, we developed heart-specific conditional knockout mice with the *ChAT* gene (hChAT KO) and observed significant effects on the cardiac mitochondria. However, they have not been corroborated or documented in previous reports. One of the distinctive and original aspects of the current study is that it focused on how the loss of NNCCS function influences intracellular organelles of cardiomyocytes and cardiac function. Our results suggest that the NNCCS or non-neuronal ACh in the heart specifically influences mitochondrial structures and functions because the heart suffers from heart-failing conditions even in a physiological situation. Therefore, the NNCCS is essential for the physiological stability and integrity of the heart.

## Methods

### Reagents and compounds

Tamoxifen (Tx: T5648), soybean oil (S7381), and mecamylamine hydrochloride (M9020) were purchased from Sigma-Aldrich. Methyllycaconitine citrate (ab120072) and GTS-21 dihydrochloride (ab120560) were purchased from Abcam (Cambridge, U.K.). PNU282987 (S5930) was purchased from Selleck Biotech (Kanagawa, Japan).

### Development of the heart-specific conditional ChAT KO mice (hChAT KO)

The best PAM sequences were searched using CRISPRdirect to identify those around exon 3 of the murine *ChAT* gene. Two candidate sites for gRNA (5′ PAM: 580 bp from exon 3, and 3′ PAM: 230 bp from the end of exon 3) were found to flank exon 3. The length of the genome between the two sites was calculated as 920 bp. Further 200 bp of the 5′ upstream from the 5′ PAM sequence was selected as the 5′ arm, whereas further 200 bp of 3′ downstream from the 3′ PAM sequence was selected as the 3′ arm. A donor single-strand DNA fragment, including a 5′ or 3′ homologous region to the 5′ or 3′ arm, exon 3 of the *ChAT* gene, and 5′ or 3′ loxP sequence in each arm flanking exon 3, was used for CRISPR-Cas 9 genome editing. Finally, homologous recombination was confirmed that two loxP sequences were inserted in both 5′ and 3′ of the *ChAT* exon 3 gene (ChAT^f/+^). Male and female mice with heterologous loxP sequences flanking the *ChAT* exon 3 gene (ChAT^f/+^) were mated to obtain homologous (ChAT^f/f^) mice. Heart-specific Tx-inducible Cre recombinase gene-expressing mice (B6.FVB(129)-A1cf^Tg(Myh6-cre/Esr1*)1Jmk/^J: IMSR_JAX:005657) were purchased from Jackson Laboratory (Bar Harbor, ME, U.S.A.) and mated with ChAT^f/f^ mice to acquire heart-specific ChAT KO (ChAT^f/f^αMHC-Cre) mice, which were confirmed to express Cre in their hearts. Moreover, using another mouse expressing the endothelial cell-specific Cre recombinase gene (Cdh5-BAC-CreERT2), generously provided by Professor Yoshiaki Kubota (Department of Anatomy, Keio University, Japan) [15], another KO mouse was obtained by mating with ChAT^f/f^ mice (ChAT^f/f^/Cdh5-Cre mice). The endothelially expressed *ChAT* exon 3 gene was deleted by Tx (eChAT KO mice), and the eChAT KO mice were compared with hChAT KO mice for the evaluation of cardiac function. For the deletion of a gene of interest in a mouse, 4 mg of Tx (T5648, Sigma-Aldrich) dissolved in 200 of μL soybean oil (Oil, S7381, Sigma-Aldrich) was incubated at 56°C with agitation until complete dissolution and injected intraperitoneally. Gene deletions were confirmed via PCR using the heart-derived genomic DNA. Deletion-induced shorter PCR products were detected using template DNA from the heart, but not from the other organ-derived DNA after Tx treatment. Finally, hChAT KO mice were generated by injecting Tx in soybean oil into ChAT^f/f^ /αMHC-Cre mice. Hereafter, Tx-induced gene-deletion mice will be referred to as Tx or hChAT KO mice. In contrast, ChAT^f/f^αMHC-Cre mice injected with soybean oil alone were used as controls and are shown as Oil or Control.

Gene deletion by Tx was confirmed to require at least three days following injection. The PCR primers used to check for the deletion of exon 3 of the murine *ChAT* gene were as follows: a 5′ forward primer: GTCAGGAGACTCTAGAGG; a 3′ reverse primer: TATGACCATGCCTTGAGG, using a PCR condition: 35 cycles of a protocol including 94°C for 30 s, 60°C for 30 s, and 72°C for 90 s. The non-deleted PCR fragment length was 1208 bp; in contrast, the deletion by Cre recombinase decreased the fragment to 337 bp.

All mice for Control Oil or KO Tx utilized in this study were male and around two months of age.

### Animal studies

An animal study using a mouse under anesthesia included only a cardiac function study with a PV system, as mentioned below. In that study, mice were anesthetized with isoflurane (0.6–1.0 vol%) at a flow rate of 0.5–1.0 L/min (NARCOBIT, Natsume Seisakusho, Co., Ltd, Tokyo, Japan). After measurement, mice were killed by cervical dislocation. In studies, other than the function study, mice were euthanized by cervical dislocation to extract interested organs.

### Western blot (WB) analysis

The whole heart (100 mg) or atrium excised from a killed mouse with cervical dislocation was homogenized in 1 ml of T-PER^TM^ (Tissue Protein Extraction Reagent, Thermo Scientific^TM^) on ice, followed by centrifugation (10,000 *g*, 4°C, 10 min) to obtain the supernatants. The protein concentration was determined using a PIERCE^TM^ BCA Protein Assay Kit. The supernatants were mixed with 6 × sample buffer to obtain a 600 μl sample in 1 × sample buffer. A sample with a comparable amount of protein (10–50 μg/lane, depending on the protein of interest) was electrophoresed and blotted onto a PVDF membrane. Comparable applied samples were confirmed by CBB staining. The membrane was subjected to a blocking procedure with 4% skim milk, followed by a reaction with a primary antibody, as follows. The primary antibodies included a goat anti-ChAT polyclonal antibody (ab) (1:2000, AB143, Merck Japan (Tokyo, Japan), a rabbit anti-Glut4 polyclonal ab (1:2000, NBP1-49533, Novus Biologicals, LLC, Centennial, CO, U.S.A.), a rabbit anti-Akt monoclonal ab (1:1000, #9272, Cell Signaling Technology, Danvers, MA, U.S.A.), a rabbit anti-pAkt monoclonal ab (1:500, #2965, Cell Signaling Technology), a rabbit anti-VEGF polyclonal ab (1:1000, #19003–1-AP, Proteintech Group, Inc. Rosemont, IL, U.S.A.), a rabbit anti-NOS1 monoclonal ab (1:1000, ab76067; Abcam), a rabbit anti-muscarinic acetylcholine receptor 2 monoclonal ab (1:1000, ab109226; Abcam), a rabbit anti-mitochondrial calcium uniporter (MCU) monoclonal ab (1:1000, #14997, Cell Signaling Technology), a rabbit anti-AChRα7ab (1:1000, sc-5544, Santa Cruz Biotechnology Inc., Dallas, TX, U.S.A.), a rabbit anti-VDAC polyclonal ab (1:1000, ab15895; Abcam), a rabbit anti-ANP polyclonal ab (1:1000, sc-18811, Santa Cruz Biotechnology Inc.), and a rabbit anti-GAPDH monoclonal ab (1:5000, #2118, Cell Signaling Technology). After incubation with the primary antibody, the membrane was incubated with a secondary antibody conjugated with horseradish peroxidase. Following the treatment with ImmunoStar® LD (Wako Pure Chemical Industries, Ltd., Tokyo, Japan), a band of an interested protein was detected by C-DiGit Blot Scanner (LI-COR Corp., Lincoln, NE, U.S.A.), as reported previously [11].

### Detection of mitochondrial genome DNA and ChAT mRNA by qPCR

This was performed referring to a manufacturer’s instruction of a mitochondrial DNA monitoring kit (TAKARA Bio Inc., Shiga, Japan). A mitochondrial copy number was measured by PCR using genomic DNA isolated from the heart, two kinds of murine-specific primers, SLCO2B1 and SERPINA1, for the nucleus and two other primers, ND1 and ND5, specific for mitochondria. The procedure and PCR conditions for mice were based on a Mitochondrial DNA (mtDNA) monitoring primer set (TAKARA Bio Inc., Shiga, Japan) and partly modified for murine use. The primers used were as follows:

mSloco2b1 5′ forward primer: GCCACCTTCCTGCCTAAGTT;

mSloco2b1 3′ reverse primer: ACGCTGAAGAGCAGACACAA;

mSERPINA1a 5′ forward primer: CCTGGGAGACTTTGCAATCA;

mSERPINA1a 3′ reverse primer: CTCCACCAGCTTCAGGTCAT;

mND1 5′ forward primer: GTTGGTCCATACGGCATTTT;

mND1 3′ reverse primer: TCTCCTTCTGTCAGGTCGAA;

mND5 5′ forward primer: CCATGCTTATCCTCACCTCAG;

mND5 3′ reverse primer: GATTTTCCTGTAGCTGCGATT.

After obtaining the heart-derived genomic DNA using NucleoSpin^®^ Tissue (TAKARA Bio Inc.), qPCR was performed using the Thermal Cycler Dice^®^ Real-Time System TP800 (TAKARA Bio Inc. Shiga, Japan). The PCR condition was 98°C for 2 min followed by 40 cycles of a protocol including 94°C for 10 s, 60°C for 15 s, and 68°C for 30 s. After PCR, the difference in ΔCt values was measured between ND1 and mSLCO2B1, or between ND5 and SERPINA1. Using each ΔCt value, 2^ΔC^ was calculated, and the average value was obtained as the copy number, according to the manufacturer’s instruction.

To perform qRT-PCR of ChAT mRNA in the heart, total RNA was isolated from the control heart of vehicle-administered hChAT KO mice and Tx-treated hChAT KO mice, both of which were euthanized with cervical dislocation, using ISOGEN II (Nippon Gene Co., Ltd., Tokyo, Japan). A 0.5 μg of total RNA was reverse-transcribed with ReverTra Ace^®^ qPCR RT Master mix (Toyobo Life Science, Osaka, Japan) to synthesize a cDNA template, which was amplified by GoTaq^®^ qPCR Master Mix (Promega KK, Tokyo, Japan) with Thermal Cycler Dice® Real-Time System TP800 (TAKARA Bio Inc.). A relative expression level of ChAT mRNA or BNP mRNA was quantified to the levels of β-actin and compared between the control heart and hChAT KO heart, using the following primers, as used in our previous study [16],

ChAT (forward): TGGATGAAACATACCTGATGA GCAA;

ChAT (reverse): CGTGAAAGCTGGAGATGCAGAA;

BNP (forward): CTGAAGGTGCTGCCCCAGATG;

BNP (reverse): GACGGATCCGATCCGGTC;

β-actin (forward): CATCCGTAAAGACCTCTATGC CAAC; and

β-actin (reverse): ATGGAGCCACCGATC CACA.

### Measurement of ACh levels in the heart

According to our previous studies, the cardiac ventricles excised from an euthanized mouse with cervical dislocation were obtained by completely excluding the atria from the whole heart, and the samples were properly processed to measure ACh levels using HPLC [10]. The heart weight and lysis buffer volume were comparable between each sample; therefore, the ACh level was expressed in mol/heart.

### Evaluation of cardiac function

Cardiac function of mice under anesthesia with isoflurane (0.6–1.0 vol%) was measured using an ADVantage PV system through an apical approach with an admittance PV catheter (1.2F; Transonic Systems Inc., Ithaca, NY, U.S.A.), as reported in our previous studies [13], under artificial ventilation conditions. Hemodynamic parameters included heart rate (HR), end-systolic pressure (ESP), end-diastolic pressure (EDP), end-systolic volume (ESV), end-diastolic volume (EDV), stroke volume (SV), cardiac output (CO), ejection fraction (EF), dP/dt min, and tau.

### Morphological examination using a transmission electron microscope

For electron microscopic analysis, the hearts of euthanized mice with cervical dislocation were perfused and fixed with 2% paraformaldehyde-2.5% glutaraldehyde solution-0.1M phosphate buffer (pH 7.4). The transverse slices of the heart were prepared and post-fixed with 1% OsO_4_-0.1M phosphate buffer. The heart was dehydrated with graded ethanol and embedded in Epon 812. Ultrathin sections were cut using an ultramicrotome and stained with uranyl acetate and lead citrate. Images were captured using a JEOL JEM-1400Plus transmission electron microscope equipped with an EM-14360 Flash CMOS Camera (JEOL Ltd., Tokyo, Japan) and analyzed.

### Evaluation of mitochondrial functions

#### Measurement of an ATP content in the heart

According to our previous study, ATP levels were measured using the ‘tissue’ ATP assay kit (TOYO B-Net, Tokyo, Japan), following the manufacturer’s instructions [16]. The heart of an euthanized mouse with cervical dislocation was homogenized in 10 ml of the homogenizing buffer, followed by dilution with 8. After isolating ATP from the heart using an isolation buffer, a portion of the sample was mixed with a luminescent reagent. The luminescence level was measured, and the ATP concentration was calculated using the standard ATP content. ATP levels are expressed as nM/g tissue.

#### Measurement of GSH/GSSG in the heart

The concentration of GSSG and total glutathione in the heart of an euthanized mouse with cervical dislocation was measured using a GSSG/GSH quantification kit (DOJINDO LABORATORIES, Kumamoto, Japan) following the manufacturer’s instructions. The GSH concentration was determined by subtracting GSSG from the total glutathione. The GSH/GSSG ratio was then calculated.

#### Western blot analysis of mitochondria-related proteins

Mitochondria were isolated from the heart of an euthanized mouse with cervical dislocation using the Mitochondria Isolation Kit for Tissue (Abcam) following the manufacturer’s instructions, and were obtained by sequential centrifugation. During this procedure, the cytosolic fraction was also obtained and used for subsequent experiments. Mitochondria were isolated from control (Oil) and hChAT KO (Tx) hearts. After protein quantification, 10 μg of cardiac mitochondria or 20 μg of cytosol fraction was subjected to western blot analysis using total OXPHOS rodent WB antibody cocktail (1:500, ab110413, Abcam), a rabbit anti-ND-1 monoclonal ab (1:1000, ab181848, Abcam), a rabbit anti-COXIV polyclonal ab (1:1000, 3638–100, BioVision or Abcam), a rabbit anti-VDAC polyclonal ab (1:1000, ab15895; Abcam), and a rabbit anti-cytochrome C monoclonal ab (1:1000, #11940, Cell Signaling Technology), a rabbit anti-Mitofusin-2 monoclonal ab (1:1000, #9482, Cell Signaling Technology), a rabbit anti-DRP1 polyclonal ab (1:1000, 12957–1-AP, Proteintech Group, Inc.), and a rabbit anti-AChRα7ab (1:1000, Santa Cruz Biotechnology Inc.). The next step after the reaction with the primary antibody was performed following the procedures described for western blot analysis.

#### Evaluation of mitochondrial membrane potential

For the analysis of mitochondrial membrane potential, tetramethylrhodamine methyl ester perchlorate (TMRM) (FUJIFILM Wako Chemicals) was used. Isolated mitochondria were stained by a reagent for membrane potential (TMRM), followed by flow cytometry analysis with BD FACSCanto II^TM^ flow cytometer system (Becton, Dickinson and Company, Franklin Lakes, NJ, U.S.A.). The percentage of each membrane potential dye above the peak was analyzed and compared between the control (Oil) and hChAT KO (Tx) groups. To examine the number of mitochondria, MitoTracker^®^ Green FM (Thermo Fisher Scientific, Waltham, MA, U.S.A.) was used for labeling of isolated mitochondria. Then, labeled mitochondria were measured using flow cytometry analysis with BD FACSCanto II^TM^ system.

#### Assay for calcium accumulation in isolated mitochondria derived from the heart

To elucidate how calcium accumulated in the mitochondria of the heart, in other words, how cardiac mitochondria handled cytosolic calcium, the isolated mitochondrial pellet from one heart of an euthanized mouse with cervical dislocation, as shown in the above section, was resuspended in 100 μL of a mitochondria-suspension buffer (10 mM HEPES, 125 mM KCl, 25 mM NaCl, 5 mM disodium succinate, 0.1 mM Pi(K), and 100 μM CaCl_2_, pH7.4), which was prepared according to a previous report [17]. Mitochondria were pretreated with 1 μM of mecamylamine hydrochloride (FUJIFILM Wako Chemicals), which is a non-specific nicotinic receptor antagonist, or methyllycaconitine citrate (Abcam), which is an α7 nAChR antagonist, for 10 min. Subsequently, Fluo 4-AM was added to the mitochondrial suspension (a final concentration: 5 μM) for 15 min. Thereafter, 1 μM ACh, an agonist of α7 nAChR, 1 μM GTS-21 dihydrochloride (Abcam), or PNU282987 (Selleck Biotech) was added and incubated for 60 min. The suspension was then centrifuged, and the mitochondrial pellet was resuspended in fresh suspension buffer. Fluorescence signals in the 96-well plates were immediately measured using a fluorescence plate reader (Infinite^®^ 200PRO; Tecan Trading AG, Switzerland).

### Evaluation of autophagy using western blot analysis

As described in the section above, proteins isolated from the heart excised from an euthanized mouse with cervical dislocation using T-PER^TM^ or from cells treated with a sampling buffer were electrophoresed and blotted onto the PVDF membrane, which was reacted with the following antibodies: a rabbit anti-LC3A/B monoclonal antibody (1:1000, #12741, Cell Signaling Technology), rabbit anti-p62 polyclonal antibody (1:1000, PM045; Medical & Biological Laboratories Co., Ltd., Tokyo, Japan), and rabbit anti-nitrotyrosine polyclonal antibody (1:1000, #06–284, Merck Japan, Tokyo, Japan). Furthermore, proteins from mitochondrial or cytosol fraction were subjected to western blot analysis using a rabbit anti-PARK2/Parkin polyclonal antibody (1:1000, #14060–1-AP; Proteintech), rabbit anti-PINK1 polyclonal antibody (1:1000, #23274–1-AP; Proteintech), rabbit anti-GAPDH monoclonal ab (Cell Signaling Technology), and rabbit anti-VDAC polyclonal ab (Abcam). Thereafter, the same procedure was conducted using an appropriate secondary antibody conjugated with horseradish peroxidase, and signals were detected.

### Cell culture

Murine fetal cardiomyocytes were isolated from 8 decapitated fetuses at full term with modified collagenase concentrations. The heart was excised and digested in a digestion buffer containing 0.1% collagenase, in which type 2 and 4 collagenases were mixed equally (Worthington Biochemical Corporation, Lakewood, NJ, U.S.A.). After 45 min of digestion at 37°C with agitation, digested cardiomyocytes were centrifuged and resuspended in a lysis buffer without collagenase, followed by filtration with a 40 μm cell strainer. After differential adhesion within 3 min, cardiomyocytes that did not attach to the culture dish were collected and cultured in DMEM F-12 with 1 × ITS (FUJIFILM Wako Chemicals) and 5% FBS for three days. Thereafter, they were further cultured in the same medium except for 2% FBS. Synchronized spontaneous beating of cardiomyocytes was usually observed four days after inoculation. The fetal cardiomyocytes were used for an immunofluorescent or an *in vitro* knockout study. When fetal cardiomyocytes with almost 100% confluency were prepared, the culture medium in each well was replaced with 500 μL of the complete medium without antibiotics but with 6 μg/ml hexadimethrine bromide (Sigma-Aldrich), followed by addition of Cre recombinase Gesicle (5 μL) (#631449, Clontech Laboratories, Inc., TAKARA Bio Inc., Shiga, Japan) to the well to delete flox-flanking *ChAT* gene [18]. The cells were incubated overnight, and thereafter, the culture medium was changed and used for further experiments. Cre-treated cardiomyocytes were identified by the fluorescence of mCherry.

Then, cardiomyocytes were loaded in 5 μM of Calbryte^TM^ 520^AM^ (AAT Bioquest, Pleasanton CA, U.S.A.) resolved in HEPES buffer with 0.04% Pluronic^®^ F-127 (Biotiumn Inc., Fremont, CA, USA) for 30 min. The cardiomyocytes were then electrically stimulated with USE-210 at a fixed frequency (1 Hz, 10 ms duration, 30 mA, UNIQUE MEDICAL, Tokyo, Japan). During stimulation, cardiomyocytes were observed under a fluorescent microscope (IXplore Standard; EVIDENT, Tokyo, Japan) connected to a DP75 digital camera (EVIDENT), together with cellSence imaging software (EVIDENT) to record calcium oscillations. The recorded movie was processed using Image J, and oscillation patterns between control cardiomyocytes and hChAT KO cardiomyocytes were compared, especially focusing on the dilation phase. Tau was evaluated by curve fit analysis of GraphPad Prism 10 software (GraphPad Software Inc., La Jolla, CA, U.S.A.).

The adult heart from euthanized mice by cervical dislocation in the thorax was perfused through the right ventricle with a perfusion buffer [1.25 mg/ml taurine, 1.01 mg/ml 2,3-butanedione oxime (BDM), 2 mM EDTA in HBSS(-)] to exclude circulating blood from the inferior vena cava. After clamping the ascending aorta, the heart was excised. The left ventricle of the clamped heart was also perfused with the perfusion buffer in a culture dish. Thereafter, it was perfused by a digestion buffer [1.25 mg/ml taurine, 1.01 mg/ml BDM, 1.26 mM CaCl_2_, 0.5 mM MgCl_2_, 0.4 mM MgSO_4_, 0.1 mg/ml dispase II, 1.0 mg/ml collagenase type 2, 1.0 mg/ml collagenase type 4 in HBSS (-)]. The finely digested heart was minced and subjected to gentle pipetting. After adding a perfusion buffer with 5% FBS, the cell suspension, subjected to cell strainers (100 μm), was centrifuged, resuspended in a perfusion buffer, and subjected to a 20 μm-cell strainer. The cardiomyocytes left on the membrane were collected and cultured on laminin (5 μg/ml PBS)-coated culture medium.

Fetal cardiomyocytes or adult cardiomyocytes were loaded with MitoTrackerTM Orange (ThermoFisher Scientific) for 20 min, followed by treatment with 4% PFA and 0.1% Triton-X. Immunocytochemical staining was conducted according to our previous study [12,19]. After blocking with 1% BSA at 37°C 10 min, cells were treated with a rabbit anti-AChRα7 ab (1:100, sc-5544, Santa Cruz Biotechnology Inc.) at 4°C overnight, followed by a reaction with donkey anti-rabbit IgG (Alexa Fluor® 488) antibody (ab150073, Abcam). Immunofluorescent signals were observed using confocal microscopy (Leica Microsystems, Wetzlar, Germany).

AC16 cells were commercially purchased and maintained in DMEM F12 with 12.5% FBS. AC16 cells were transfected with a ChAT-specific siRNA SMARTpool, which was commercially validated or scrambled siRNA (Dharmacon^TM^ Reagents), using DharmaFECT 1 Transfection Reagent (Horizon Discovery, Cambridge, U.K.). The response to autophagy and impairment of mitochondrial function was evaluated using autophagy antibodies.

### Evaluation of plasma HMGB-1 and IL-6 levels

Plasma levels of HMGB-1 (Arigo Biolaboratories Corp., Zhubei City, Taiwan) or IL-6 (proteintech^®^) of euthanized mice with cervical dislocation were measured by each ELISA kit, according to each manufacturer’s instructions

### Evaluation of the blood–brain barrier function and influences on depression or anxiety-like behavior

The brain from an euthanized mouse with cervical dislocation was excised, and the expression levels of the blood–brain barrier (BBB) components, claudin-5 and ZO-1, were evaluated by western blot analysis, as described in the above section. As previously reported [19], following cold brain injury of an anesthetized mouse with isoflurane, 4% Evans blue dye (200 μl) was injected systemically. After confirming that the blue dye was distributed throughout the whole body, the brain was excised after cervical dislocation, and Evans blue leakage was observed. A 1 mm slice of the brain was sectioned to cover the leakage area. The leaked section was soaked in 500 μL of formamide and incubated at 56°C for one day. Thereafter, the absorbance at 600 nm was measured, and the leakage level was compared between control and hChAT KO mice.

The tail suspension test (TST) was conducted as follows: The mice were suspended in the tails at a height of 25 cm from the floor for 10 min. The immobilization time spent in the test was measured and compared as previously reported [11]. Another test was performed using the elevated plus maze test (EPMT), according to our previous study [11]. A mouse was placed in the center of the EPM facing an open arm; thereafter, the time spent in an open arm was measured within 10 min.

### Statistical analysis

Statistical analyses were performed using GraphPad Prism 10 software for Windows (GraphPad Software Inc., La Jolla, CA, U.S.A.). They included the unpaired *t-test* or Mann−Whitney test for two-group comparisons, and for multiple comparisons, more than two groups were analyzed using one-way analysis of variance (ANOVA), followed by Tukey’s multiple comparison test. Data are expressed as mean ± SD in two-group comparisons; otherwise, data are presented as mean ± SEM. Statistical significance was set at *P* value of <0.05.

## Results

### Characteristics of hChAT KO mice

**Figure 1:**
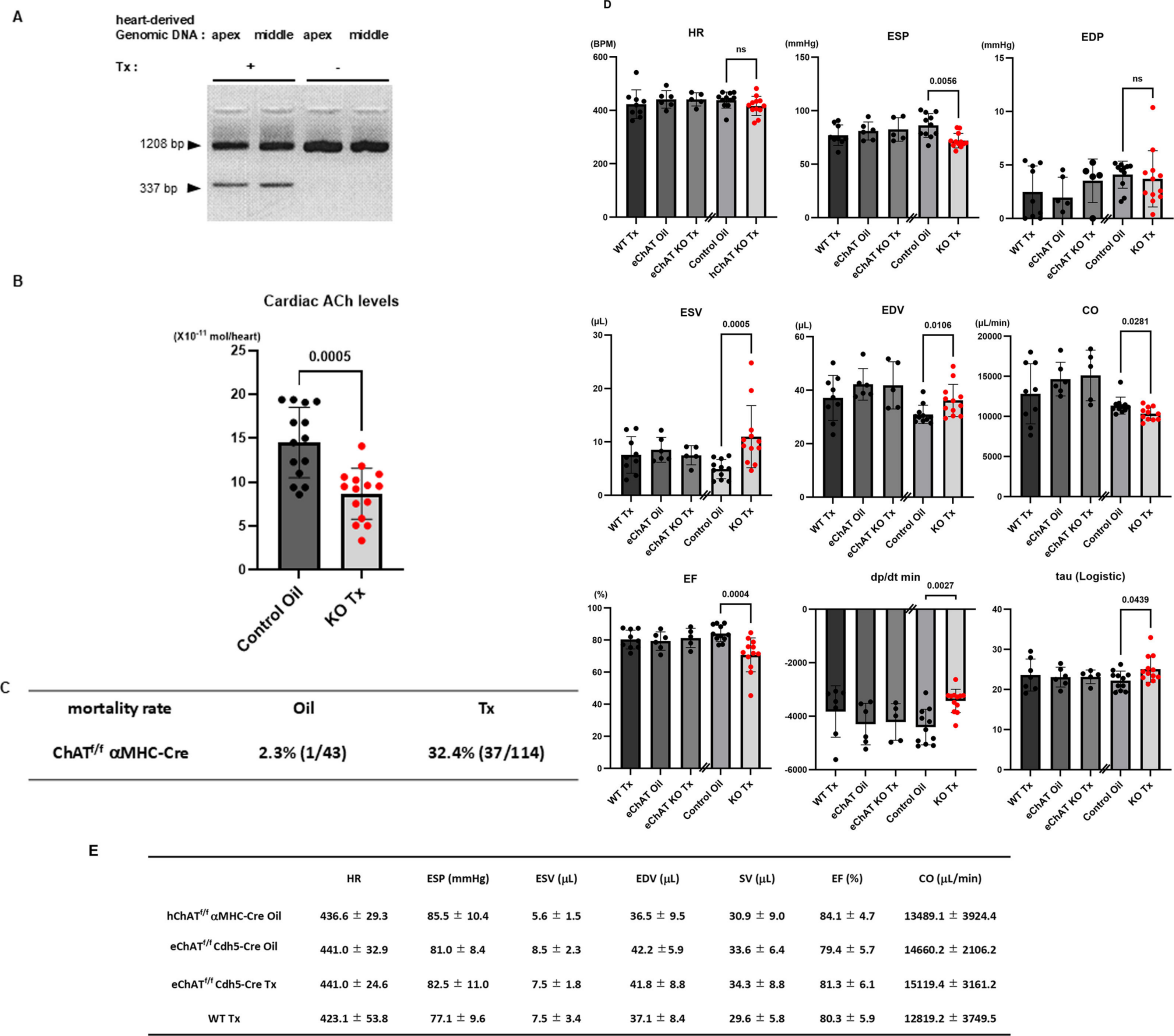
ChAT gene deletion by Tx-mediated cre induction specifically in the heart (hChAT KO mice), identified by down-regulated cardiac ACh levels, leading to cardiac dysfunction, which is partly associated with heart failure-like phenotypes. **A**. Gene deletion by Cre was confirmed by PCR using a deleted DNA fragment (337 bp). **B**. Cardiac ACh levels were significantly decreased in hChAT KO mice (KO Tx vs. Control oil, *P*<0.01). **C**. The mortality rate of hChAT KO mice (Tx) was higher than that of control mice (Oil). **D**. Hemodynamic parameters were significantly altered in hChAT KO mice (KO Tx), as evidenced by the significant down-regulation of ESP (*P*<0.01), CO (*P*<0.05), and EF (*P*<0.01), and reciprocally, by the significant up-regulation of ESV (*P*<0.01), EDV (*P*<0.05), -dP/dt (*P*<0.01), and tau (*P*<0.05). ns, not significant. **E**. Tx did not affect cardiac function parameters in eChAT KO and WT mice, although Tx-treated hChAT KO mice suffered from impaired cardiac dysfunction, as shown in D.

**Figure 2:**
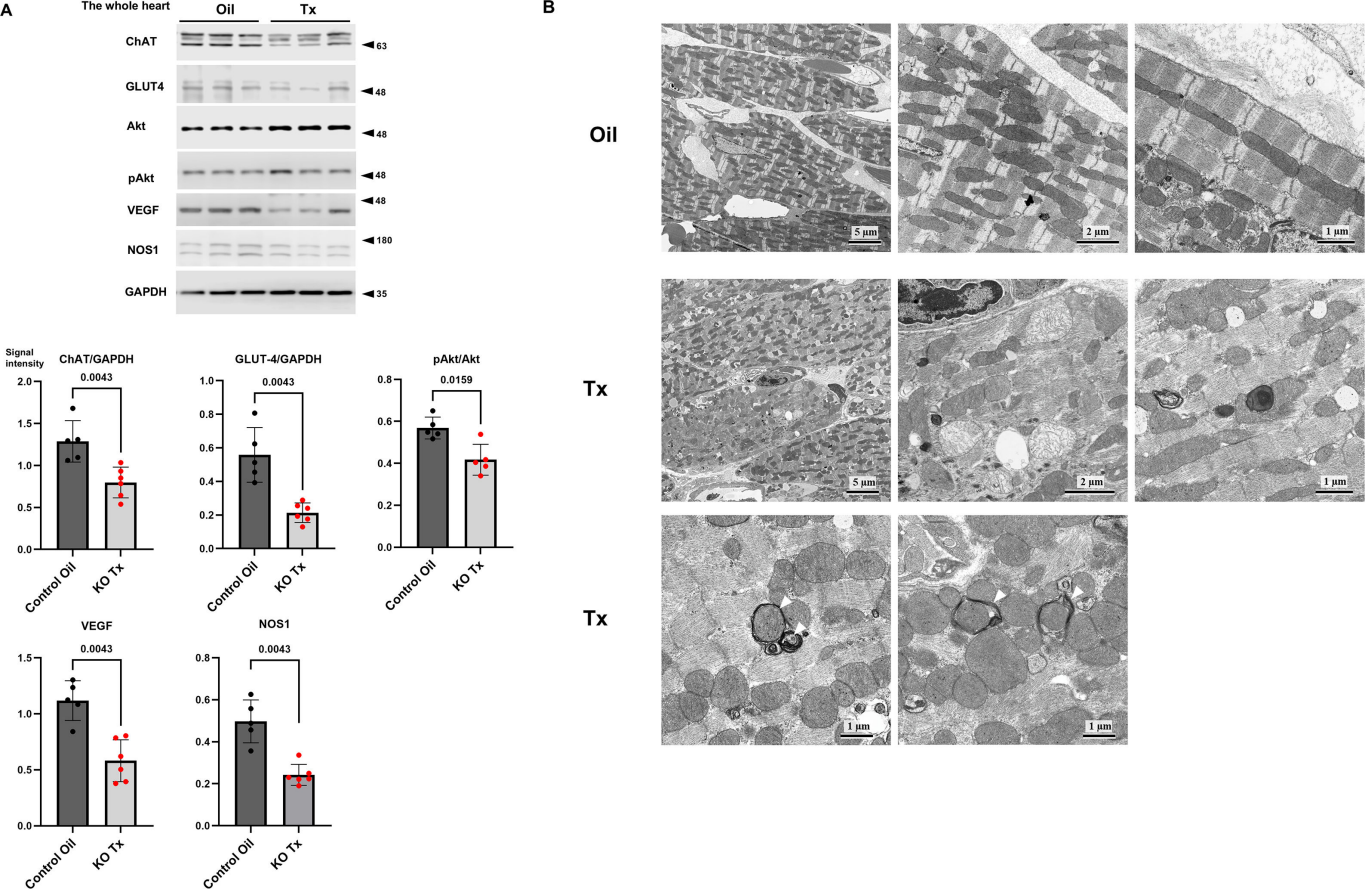
Mitochondrial dysfunction in hChAT KO mouse hearts leading to morphological changes and reduced expression levels of proteins related to glucose metabolism. **A**. Gene deletion significantly decreased the ChAT protein levels in the heart (*P*<0.01). The down-regulation of the NNCCS diminished levels of GLUT4 (*P*<0.01) and pAkt/AKT (*P*<0.05), and expression levels of VEGF (*P*<0.01) and NOS1 (*P*<0.01). **B**. Transmission electron microscope revealed smaller and round-shaped mitochondria associated with deranged cardiac muscle fibers (Tx) in the KO mouse heart, compared with longitudinal mitochondria found between muscle fibers (Oil) in the control mouse heart. Several mitochondria appeared with disrupted cristae structures (center panel in Tx) or were surrounded by multiple membrane structures in hChAT KO mouse hearts (white arrow heads in Tx of the lower panel). Scale bars 1.0, 2.0, or 5.0 μm.

At seven days after Tx injection (Tx+ in Figure 1A), heart-derived genomic DNA obtained from ChAT^f/f^αMHC-Cre mice showed a deleted fragment (337 bp) of the PCR product against a fragment flanking exon 3 of *ChAT* gene (1208 bp). In contrast, ChAT^f/f^αMHC-Cre mice injected with Tx vehicle (soybean oil) did not show *ChAT* gene deletion (Tx– in Figure 1A). In this study, Tx-treated ChAT^f/f^αMHC-Cre mice were hereafter considered to be hChAT KO mice, presented as ‘KO Tx or Tx’, whereas soybean oil-treated mice were deemed to be control mice, presented as ‘Control Oil or Oil’. Deletion of exon 3 of the *ChAT* gene by Tx in ChAT^f/f^αMHC-Cre mice resulted in a decreased level of ChAT protein expression in the heart (KO Tx) compared with that in control ChAT^f/f^αMHC-Cre mice injected with oil (Control Oil) (Figure 2A), whereas ChAT protein levels in the other organs including the brain, vasculature, and skeletal muscle, were comparable (Supplementary Data S1). Therefore, KO mice (KO Tx) had a reduced ACh content in the heart compared with control mice (Control Oil) [8.6 ± 3.0 vs 14.5 ± 4.0 ( × 10^-11^ mol), *n* = 14–15 each, *P*=0.0005; Figure 1B]. These results indicated that hChAT KO mice possessed significantly decreased ACh levels in the heart; therefore, the NNCCS was confirmed to be attenuated in hChAT KO mice.

### hChAT KO mice with cardiac dysfunction

After Tx injection, some of the hChAT KO mice gradually lost body weight, which was associated with symptoms of respiratory distress, that is, tachypnea, which was evident within two weeks. Their symptoms revealed that they suffered from heart failure, and thereafter, they died. These phenomena were associated with increased BNP mRNA and ANP protein levels (Supplementary Data S2). The mortality rate of hChAT KO mice was 32.4% (37 mice died among 114 Tx-injected mice, Tx) compared with 2.3% in control mice (one mouse died among 43 oil-injected mice, Oil) (Figure 1C). This strikingly higher mortality rate in KO mice suggests that the NNCCS is essential for maintaining cardiac function.

Consequently, the cardiac function of hChAT KO mice (KO Tx) was evaluated within two weeks of injection using an admittance PV catheter system and compared with that of control mice (Control Oil) (Figure 1D) and Tx-injected wildtype (WT) and eChAT KO mice (Figure 1E). The cardiac function of hChAT KO mice was impaired (Figure 1D), as ESP was significantly decreased (72.0 ± 6.8 mmHg vs. 86.2 ± 11.1 mmHg, *n* = 11–12, *P*=0.0056), which was also associated with the significant decrease in EF (70.8±10.6% vs. 84.1±5.0%, *n* = 11–12, *P*=0.0004), and CO (10421.8 ± 836.8 μL vs. 11350.2 ± 1056.3 μL, *n* = 11–12, *P*=0.0281); however, HR in hChAT KO mice was comparable with that in control mice. On the other hand, ESV (11.0 ± 5.8 μl vs. 4.9 ± 1.8 μl, *n* = 11–12, *P*=0.0005) and EDV (36.2 ± 6.0 μl vs. 30.9 ± 3.5 μl, *n* = 11–12, *P*=0.0106) were significantly increased, associated with the significant increase in -dp/dt (-3431.3 ± 436.6 mmHg/s vs. -4407.4 ± 667.0 mmHg/s, *n* = 11–12, *P*=0.0027) and tau (Logistic) (25.1 ± 3.3 msec vs. 22.2 ± 2.3 msec, *n* = 11–12, *P*=0.0439). These hemodynamic parameters indicate that the hearts of KO mice were subjected to systolic dysfunction. Moreover, since both dp/dt min and tau, both of which are diastolic function parameters, were significantly influenced, the alteration suggested that diastolic function was also impaired.

To further confirm whether the impaired cardiac function in hChAT KO mice induced by Tx contributed to the disruption of the NNCCS, but not Tx itself, WT mice and eChAT KO mice were injected with Tx, and their cardiac function was evaluated. Tx did not influence cardiac function in WT or eChAT KO mice (Figure 1E), and the cardiac function of eChAT KO mice did not significantly differ from oil-injected ChAT^f/f^/Cdh5-Cre mice, indicating that Tx did not impair heart function in WT and eChAT KO mice. The cardiac phenotype of hChAT KO mice contributes to the down-regulation of NNCCS because of *ChAT* gene deletion in the heart.

### Mechanisms underlying the impaired cardiac function of hChAT KO mice

Based on our previous studies [16], the protein expression levels of key factors in energy metabolism, angiogenesis, and nitric oxide were evaluated by western blot analysis to investigate whether down-regulation of the NNCCS influences the factors (Figure 2A). *ChAT* gene deletion significantly down-regulated the ChAT protein expression in the heart (*n* = 5–6, *P*=0.0043). Similarly, the GLUT4 levels (*n* = 5–6, *P*=0.0043) and p-AKT/AKT ratio (*n* = 5, *P*=0.0159) significantly decreased, and the expression levels of VEGF (*n* = 5–6, *P*=0.0043) and NOS1 (*n* = 5–6, *P*=0.0043) were significantly reduced in the hearts of KO mice. These results suggest that hChAT KO mice had impaired cardiac energy metabolism, specifically glucose metabolism, attenuated angiogenic potency, and blunted nitric oxide synthesis in the heart, which further supports our previous results [16].

### Morphological analysis of the hearts of KO mice

Transmission electron microscopy analysis was conducted to further investigate whether the morphological effects on intracellular organelles were induced by the down-regulation of NNCCS in control oil-treated (Oil) and hChAT KO mice (Tx) (Figure 2B). In the control heart (Oil), cardiac muscle fibers were regularly oriented with the proper appearance of the A- and Z-bands. Intact mitochondria possessing proper cristae structures were located longitudinally between the cardiac muscle fibers (center and right panels in Oil; Figure 2B). These normal structures were also observed in the hearts of the Tx-treated WT mice (data not shown). In contrast, hChAT KO hearts (Tx) partially demonstrated deranged and disorganized cardiac muscle fibers with smaller, more fragmented, and swollen mitochondria (center and right panels of Tx; Figure 2B). Close to the fibers, vacuole-like structures were observed; however, the appearance was not compatible with endothelial cells due to the lack of nuclear and cytoplasm-like structures, and the normal structures of the A-and Z-bands appeared obscure owing to probable degradation (right panel in Tx). In the magnified view, several mitochondria appeared swollen, and normal cristae structures were disrupted (center panel in Tx). Furthermore, some mitochondria appeared to be surrounded by multiple membrane structures (arrowheads in the bottom panel of Tx). These results indicate that hChAT KO mouse hearts did not maintain normal cardiac fibers, and their mitochondria were extensively damaged. Therefore, the down-regulation of NNCCS may be related to mitochondrial dysfunction in terms of function and morphology.

### Mitochondrial dysfunction of the hearts of hChAT KO mice

**Figure 3:**
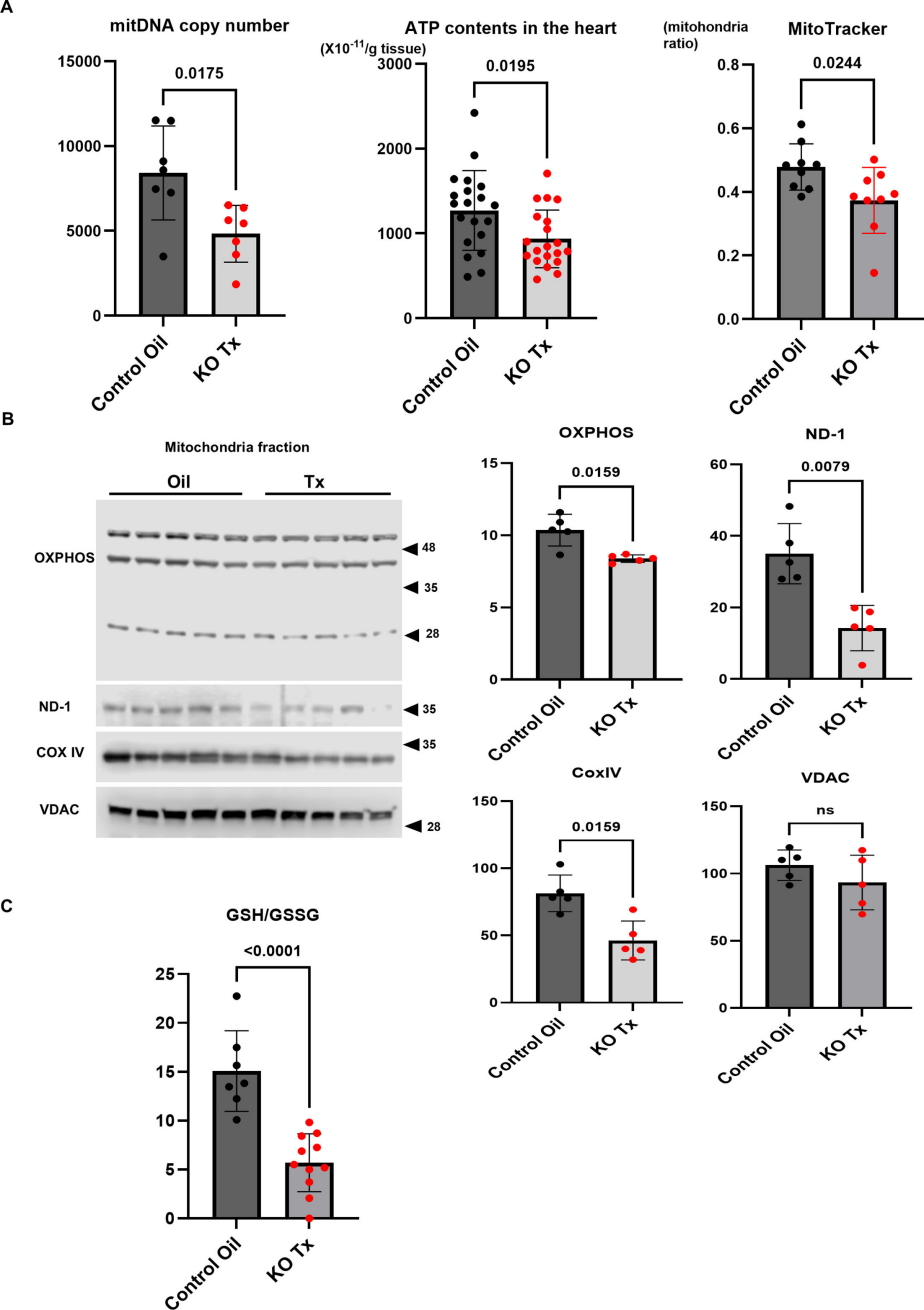
Mitochondria of hChAT KO mouse hearts are decreasing the DNA copy number, ATP production due to the diminishing respiratory electron transport chain-related proteins, and MitoTracker-staining proportion. **A**. Mitochondria-derived DNA copy number, cardiac ATP levels, and MitoTracker-positive ratio were both significantly decreased in hChAT KO mice (KO Tx) (*P*<0.05 each). **B**. In mitochondria isolated from hChAT KO mouse hearts (KO Tx), protein expression levels of a part of OXPHOS antibody-detected proteins, ND-1, and COXIV were significantly diminished (*P*<0.05, *P*<0.01, and *P*<0.05, respectively). **C**. The ratio of GSH/GSSG was significantly decreased in hChAT KO mouse hearts (*P*<0.01), indicating more ROS exposure (KO Tx).

The effects on mitochondrial parameters were evaluated in hChAT KO mouse hearts and compared with those in control mouse hearts (Figure 3A). First, the mitochondrial DNA copy number was significantly decreased in KO mice (KO Tx) (4828 ± 1672 vs 8415±2770, *n* = 7, *P*=0.0175). Therefore, ATP levels in the heart of that KO mice (KO Tx) were also significantly decreased, compared with control hearts (Control Oil) [936.2 ± 340.9 ( × 10^-11^ mol/g tissue) vs. 1271.6 ± 469.2 ( × 10^-11^ mol/g tissue), *n* = 20, *P*=0.0195). Third, the ratio of MitoTracker^®^ green FM-stained mitochondria to the whole mitochondria was significantly reduced in KO mice (0.373 ± 0.104 vs 0.478 ± 0.072, *n* = 9, *P*=0.0244) (Figure 3A). Western blot analysis of mitochondrial fractions from both hearts showed reduced expression levels of proteins identified by a total OXPHOS antibody cocktail (*n* = 5, *P*=0.0159), ND-1 antibody (*n* = 5, *P*=0.0079) and COXIV antibody (*n* = 5, *P*=0.0159) in Tx. These results suggest that mitochondrial dysfunction was associated with mitochondrial loss (Figure 3B). Based on these results, the GSH/GSSG ratio, a marker related to reactive oxygen species (ROS), in hChAT KO mouse hearts (KO Tx) was significantly decreased compared with that in control hearts (Control Oil) (6.18 ± 3.30 vs 15.07 ± 4.12, *n* = 7–11, *P*<0.0001; Figure 3C). These results suggest that the mitochondria are one of the targets of NNCCS down-regulation.

### Enhancement of autophagy in hChAT KO mouse hearts

**Figure 4:**
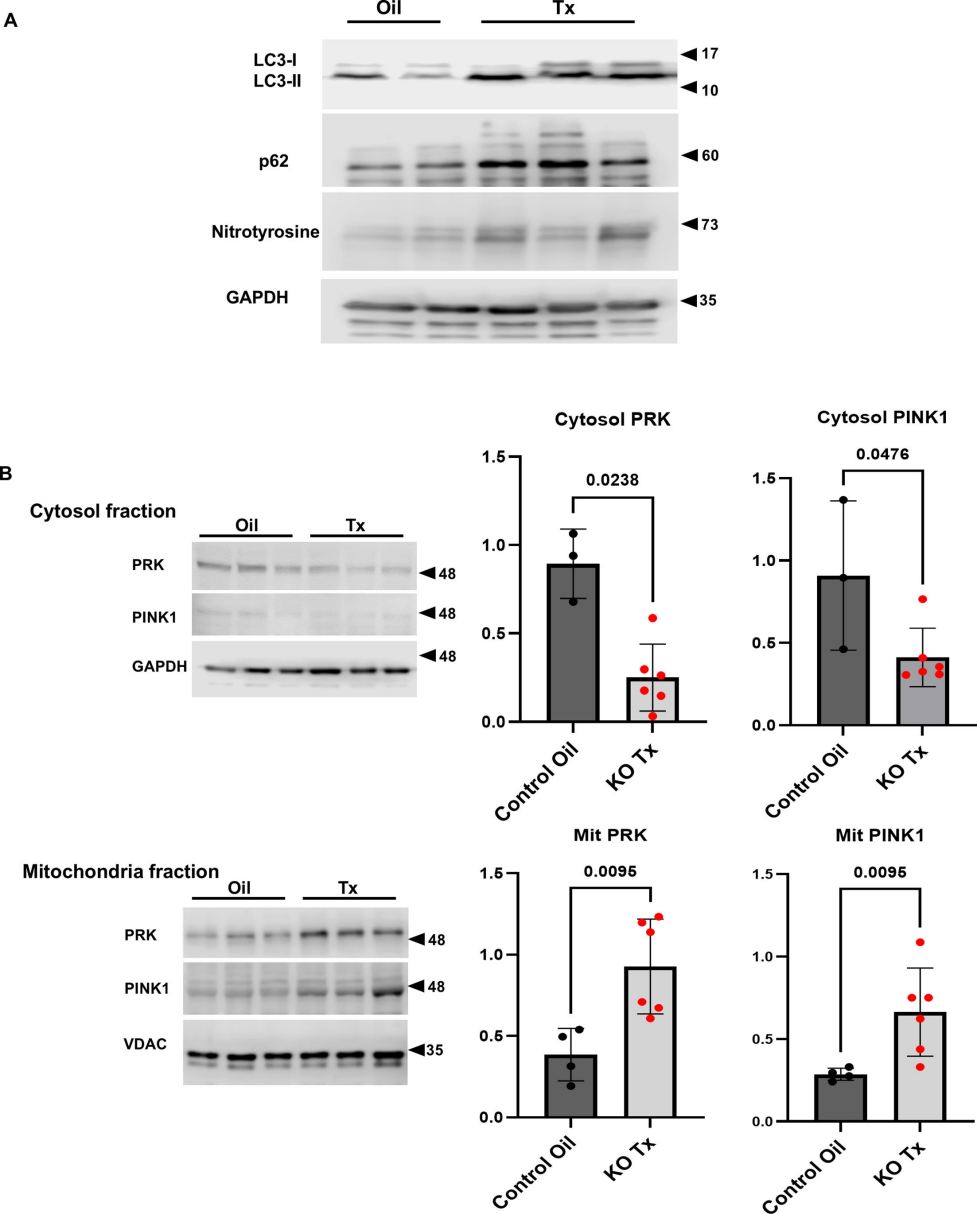
The enhancement of autophagic responses in the hearts and mitochondria in hChAT KO mice. **A**. LC3-II or LC3-I/II ratio was altered in hChAT KO mouse hearts, associated with the up-regulation of p62 and nitrotyrosine protein expression. **B**, Protein levels of both Parkin (PRK) and PINK1 were altered reciprocally between cytosolic and mitochondrial fractions of hChAT KO mouse hearts, compared with control mouse hearts. The hChAT KO reduced PRK and PINK1 protein levels in the cytosolic fraction (*P*<0.05 and *P*<0.05, respectively), however alternatively, their levels both increased in the mitochondrial fractions (*P*<0.01 and *P*<0.01, respectively).

The expression levels of LC3-I and II, and p62 were evaluated by western blot analysis. Compared with the controls (Oil), LC3-I and II levels in the Tx were altered in hChAT KO hearts (Tx) (Figure 4A). This was associated with increased p62 expression in the heart of hChAT KO mice. These results suggest that the malfunctioning mitochondria caused by NNCCS down-regulation could be increased and removed from the malfunctioning heart by inducing autophagy. Simultaneously, nitrotyrosine expression levels in the hearts were higher in hChAT KO mice (Tx) than in control mice (Oil), suggesting that the burden of nitrosative stress due to the down-regulation of NNCCS-related mitochondrial dysfunction became more evident (Figure 4A).

Altered localization of Parkin (PRK) and PINK1 was investigated between the cytosolic and mitochondrial fractions in the hearts of hChAT KO (Tx) and control mice (Oil). In the cytosolic fractions of hearts, the protein levels of PRK and PINK1 in hChAT KO mice (Tx) were decreased compared with those in control mice (Oil) (PRK: 0.251 ± 0.189 vs. 0.895 ± 0.196, *n* = 3–6, *P*=0.0238; PINK1: 0.412 ± 0.178 vs. 0.908 ± 0.453, *n* = 3–6, *P*=0.0476); however, in the mitochondria fractions of the KO mouse hearts (KO Tx), both protein levels were reciprocally increased compared with the control (Oil) (PRK: 0.926 ± 0.291 vs. 0.386 ± 0.161, *n* = 4–6, *P*=0.0095; PINK1: 0.663 ± 0.267 vs. 0.286 ± 0.037, *n* = 4–6, *P*=0.0095). These results suggest that dysfunctional mitochondria caused by NNCCS down-regulation are subjected to the autophagy-mediated induction and cleared from cardiomyocytes, indicating that cardiomyocytes respond to malfunctioning mitochondrial stress from the viewpoint of mitochondrial quality control.

### Reduction of mitochondria with normal mitochondrial membrane potential in the hearts of hChAT KO mice

**Figure 5:**
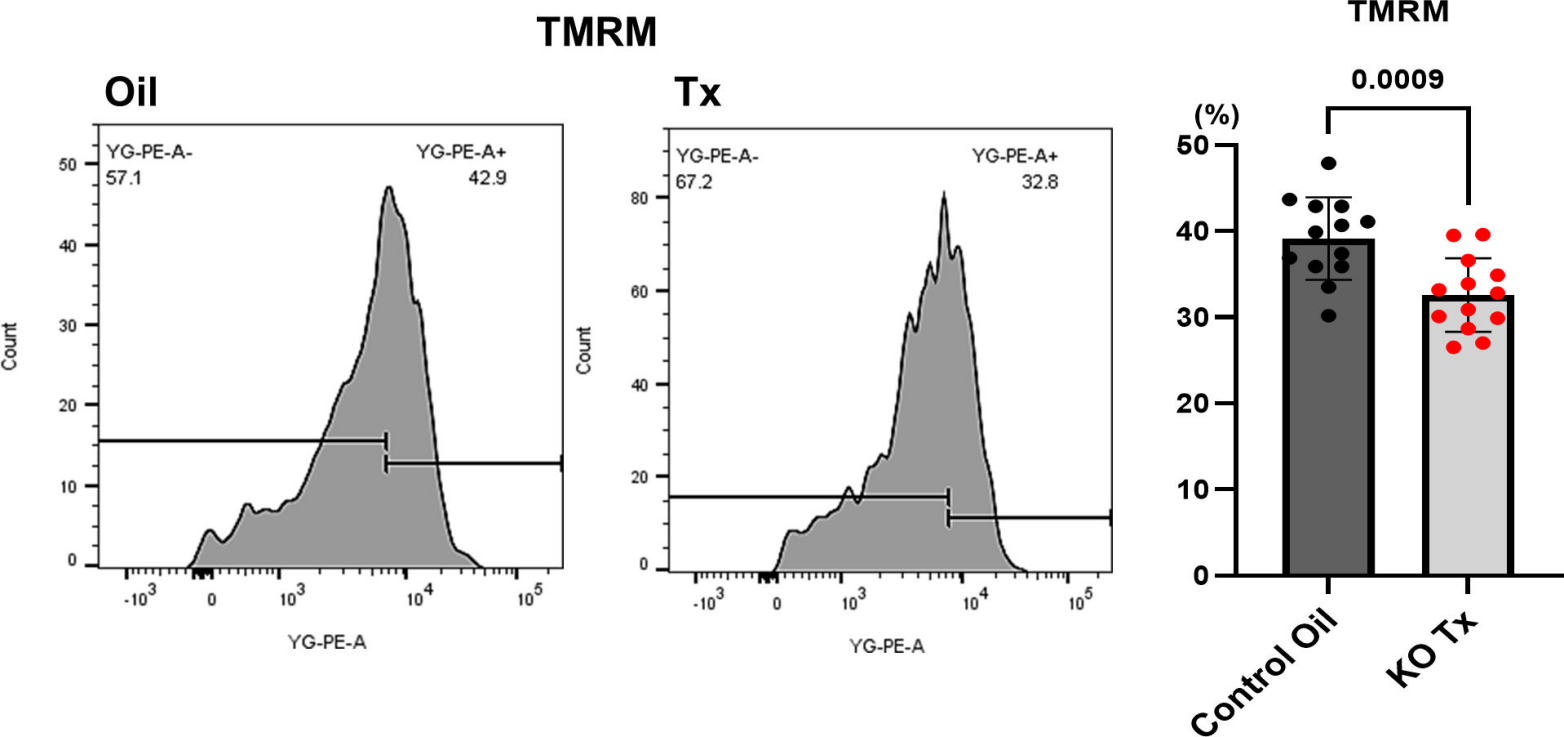
Mitochondrial membrane potential down-regulated in hChAT KO mouse hearts. The dye evaluating mitochondrial membrane potentials, TMRM, showed that mitochondria from hChAT KO hearts (KO Tx) possessed significantly a smaller proportion of the membrane potential levels over the peak than control hearts (Control Oil) (*P*<0.01).

To further evaluate mitochondrial function, the membrane potential of mitochondria isolated from hChAT KO mouse hearts was measured using a fluorescent dye, TMRM. The distribution of membrane potential levels represented by the signal intensity of the dye exhibited a broad spectrum with a peak (Figure 5). The proportion of the intensities greater than the peak of TMRM was significantly lower in hChAT KO mice (Tx) than in control mice (Oil) (32.6±4.3% vs. 39.1±4.8%, *n* = 13, *P*=0.0009). The result indicates that hChAT KO mouse hearts possess more malfunctioning mitochondria with significantly reduced membrane potential.

**Figure 6:**
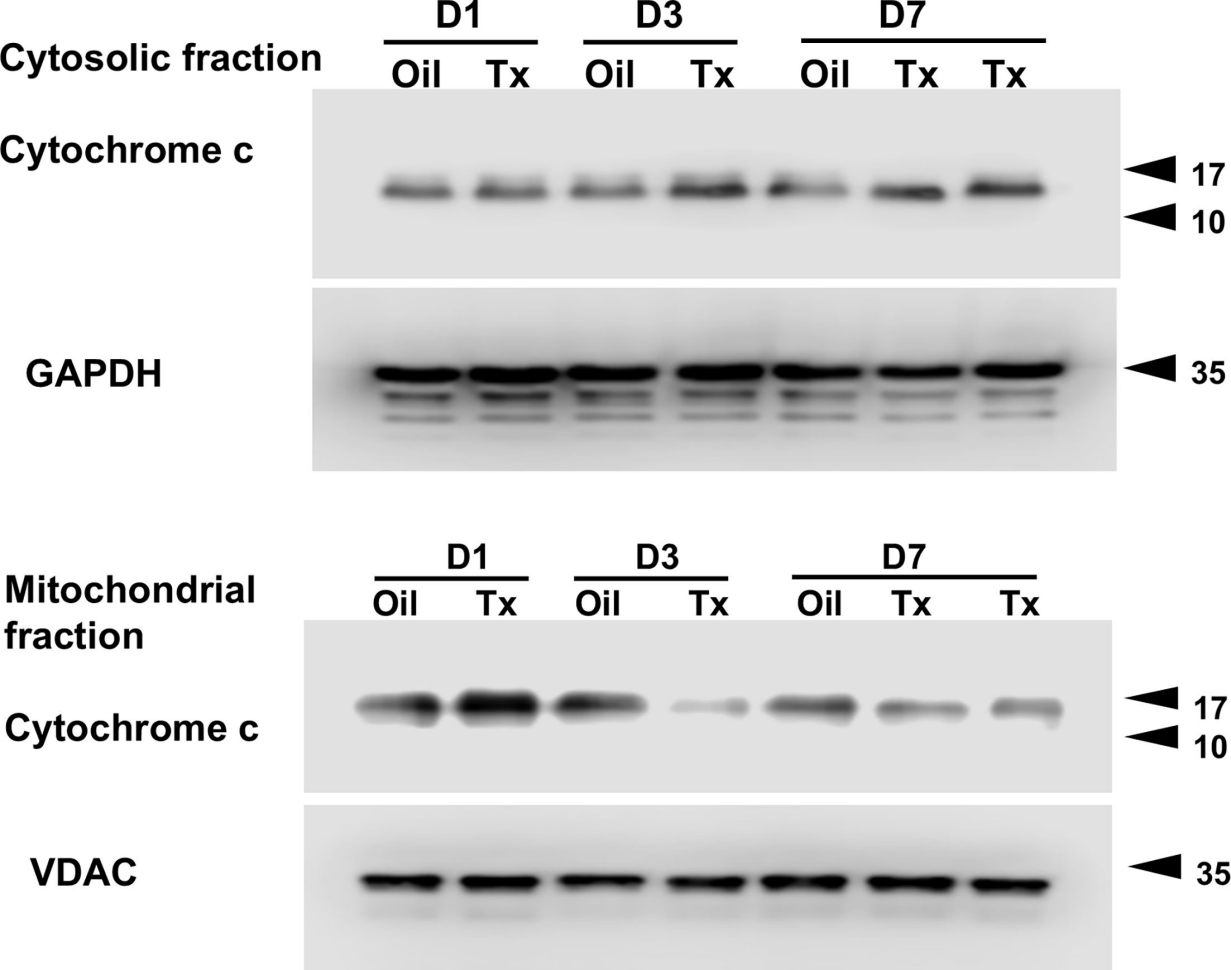
The cytochrome C release enhanced from the mitochondria to the cytosol in hChAT KO mouse hearts. Western blot analysis showed that cytochrome C protein levels were decreased in the mitochondrial fractions of hChAT KO mouse hearts from day 3 after Tx injection. In contrast, the levels in the cytosolic fractions were reciprocally increased from day 3, demonstrating that cytochrome c was released from the mitochondria to the cytosol following *ChAT* gene deletion.

In the hearts of hChAT KO mice, cytochrome c levels in the cytosolic fraction were increased after Tx injection (D3 and D7 in Tx; Figure 6). In contrast, those in the mitochondrial fractions decreased reciprocally (D3 and D7 in Oil; Figure 6). This indicates that more cytochrome c is released from the mitochondria into the cytosol in the hearts of hChAT KO mice, which also supports the finding of impaired mitochondrial function.

### α7 nicotinic ACh receptor (nAChR) expressed in the mitochondria

**Figure 7:**
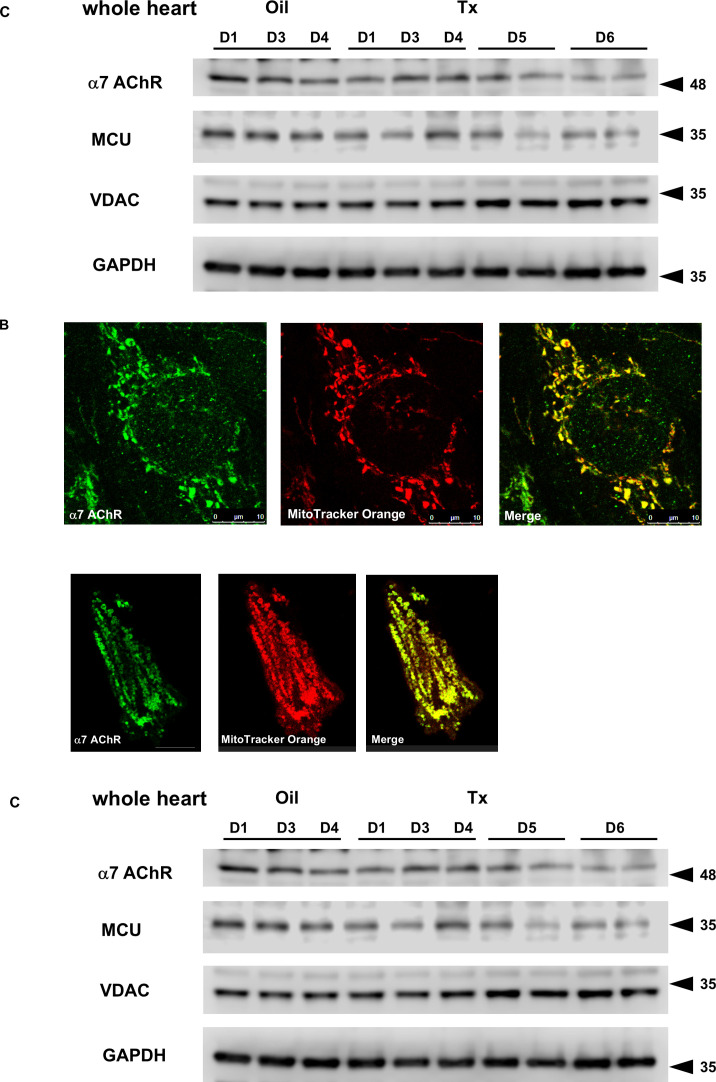
Attenuated expression of α7 nAChR, predominantly expressed in the mitochondria of the heart, and MCU in hChAT KO mouse hearts. **A**. Compared with cytosolic fractions of WT heart, mitochondrial fractions predominantly expressed α7 nAChR proteins as well as VDAC proteins, contrasting M_2_ R proteins, which were detected in the cytosol. **B**. Localization of α7 nAChR signals in the murine fetal and adult WT cardiomyocytes was overlapped with that of the mitochondrial membrane potential dye, MitoTracker Orange, suggesting that a part of α7 nAChR was expressed in mitochondria. Scale bar: 10 μm in fetal hearts (upper panels), 30 μm in adult hearts (lower panels). **C**. With *ChAT* gene deletion, protein levels of MCU and α7 nAChR in the hearts were down-regulated.

The mitochondrial and cytosolic fractions of WT hearts were subjected to western blot analysis using antibodies against α7 nAChR, muscarinic receptor (M_2_R), VDAC, and GAPDH (Figure 7A). Compared with the cytosolic fractions, α7 nAChR was expressed predominantly in the mitochondria. The differentiation between the cytosol and mitochondria was validated by the different expression of M_2_R and GAPDH in the cytosol, in contrast to VDAC in the mitochondria (Figure 7A).

Immunofluorescence staining analysis of primary cultured murine fetal cardiomyocytes also supported the predominant localization of α7 nAChR on the mitochondria (upper panels in Figure 7B). MitoTracker Orange (red), a mitochondrial membrane potential marker, indicated the localization of mitochondria around the nucleus in cardiomyocytes. Furthermore, α7 nAChR signals (green) were detected in the mitochondria (merged image in Figure 7B); nonetheless, some signals did not correspond to those of MitoTracker Orange. Similarly, in adult cardiomyocytes, α7 nAChR signals almost overlapped with those of MitoTracker Orange.

The time course of alterations in the protein expression levels of α7 nAChR and MCU was evaluated in whole-heart extracts after Tx injection (Figure 7C). The MCU plays a role in the uptake of cytosolic calcium into mitochondria to regulate calcium homeostasis in the cytosol, together with sarcoplasmic reticulum Ca^2+^ ATPase. Compared with the control hearts (Oil), the expression levels of both proteins were gradually attenuated from the fifth day (D5 and D6 in Tx). Reduced expression of α7 nAChR and MC may contribute to the loss of intact mitochondria, contrasting the VDAC levels, which were not significantly influenced in hChAT KO mice. This suggests that both proteins are susceptible to mitochondrial dysfunction. The loss of these proteins may further disturb calcium regulation in the cytosol of cardiomyocytes.

### Responses of isolated mitochondria to ACh and an α7 nAChR agonist GTS-21 and PNU282987

**Figure 8:**
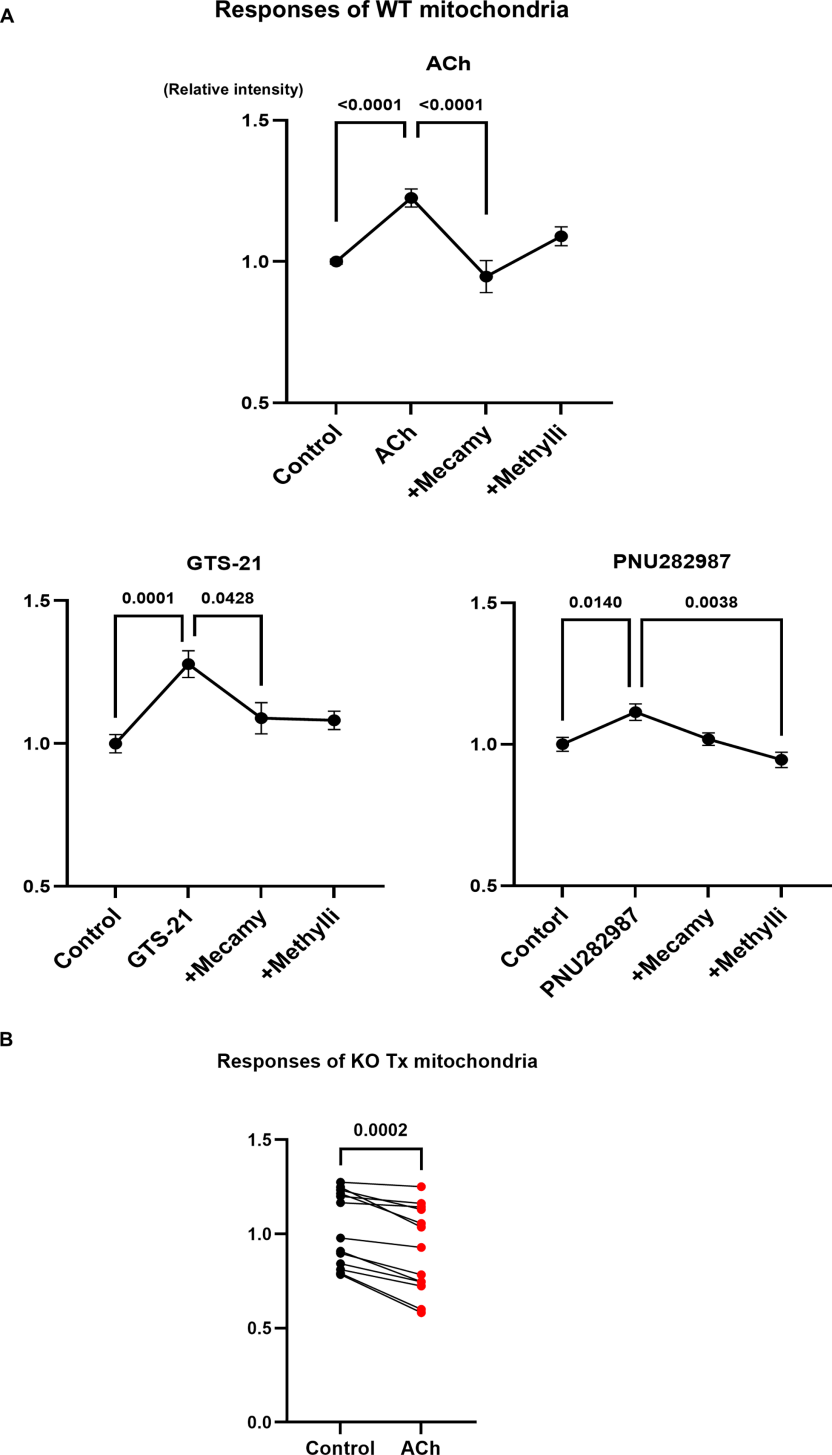
ACh or α7 nAChR agonist-mediated mitochondrial calcium accumulation through nAChRs is blunted in mitochondria of hChAT KO mouse hearts. **A**. ACh or α7 nAChR agonists (GTS-21 and PNU282987) stimulated mitochondrial calcium signals in WT mouse hearts (*P*<0.01, *P*<0.01, and *P*<0.05, respectively). Those up-regulated mitochondrial calcium accumulation was significantly blunted by mecamylamine (ACh: *P*<0.01, GTS-21: *P*<0.05) or methyllycaconitine (PNU282987: *P*<0.01). **B**. Mitochondria from hChAT KO mouse hearts did not up-regulate mitochondrial calcium signals in response to ACh (*P*<0.01).

Given that α7 nAChR was identified in the mitochondria in cardiomyocytes and that mitochondria are responsible for calcium handling in the cytosol through the MCU, isolated mitochondria from WT mouse hearts were used to evaluate the role of nAChRs in accumulating calcium using the calcium indicator Fluo-4. Isolated mitochondria pretreated with or without an antagonist, followed by Fluo-4 loading, were stimulated for 60 min with 1 μM ACh or α7 nAChR agonists GTS-21 and PNU282987. Calcium signal intensity in the mitochondria was significantly increased in both the ACh (1.23 ± 0.03 vs. 1.00 ± 0.01, F(3,34)=16.52, *P*<0.0001) and GST-21 (1.28 ± 0.04 vs. 1.00 ± 0.03, F(3,25)=9.30, *P*=0.0001) (Figure 8A) groups. The other α7 nAChR agonist, PNU282987 (1.10 ± 0.03 vs. 1.00 ± 0.02, F(3, 26) = 6.30, *P*=0.0140), also significantly accelerated calcium accumulation in the mitochondria. This suggests that calcium efficiently enters the mitochondria with the stimulation of nAChRs including α7 nAChR, which may be expressed on the outer membrane of the mitochondria. In the presence of either mecamylamine, a non-specific nAChR antagonist, or methyllycaconitine, an α7 nAChR antagonist, the accumulation of calcium in mitochondria was significantly suppressed (ACh + mecamy vs. ACh: 0.96 ± 0.10 vs. 1.23 ± 0.03, *P*<0.0001; GST-21 + mecamy vs. GST-21: 1.09 ± 0.05 vs. 1.28 ± 0.04, *P*=0.0428; PNU282987 + methylli vs. PNU282987: 0.99 ± 0.05 vs. 1.10 ± 0.03, *P*=0.0038, respectively) (Figure 8). These results suggest that mitochondrial nAChRs, including α7-nAChR, are involved in calcium entry into the mitochondria.

In contrast, when using hChAT KO mice-derived mitochondria, the accumulated calcium intensity was not elevated by ACh, but rather significantly decreased (0.93 ± 1.17 vs. 1.03 ± 1.20, *n* = 13, *P*=0.0002) (Figure 8B), suggesting that calcium entry into mitochondria or accumulation is impaired, probably due to the loss or impairment of intact mitochondria in the hearts of hChAT KO mice.

### Decay of calcium spike impaired in cardiomyocytes of ChAT^f/f^ mice treated with Cre

**Figure 9:**
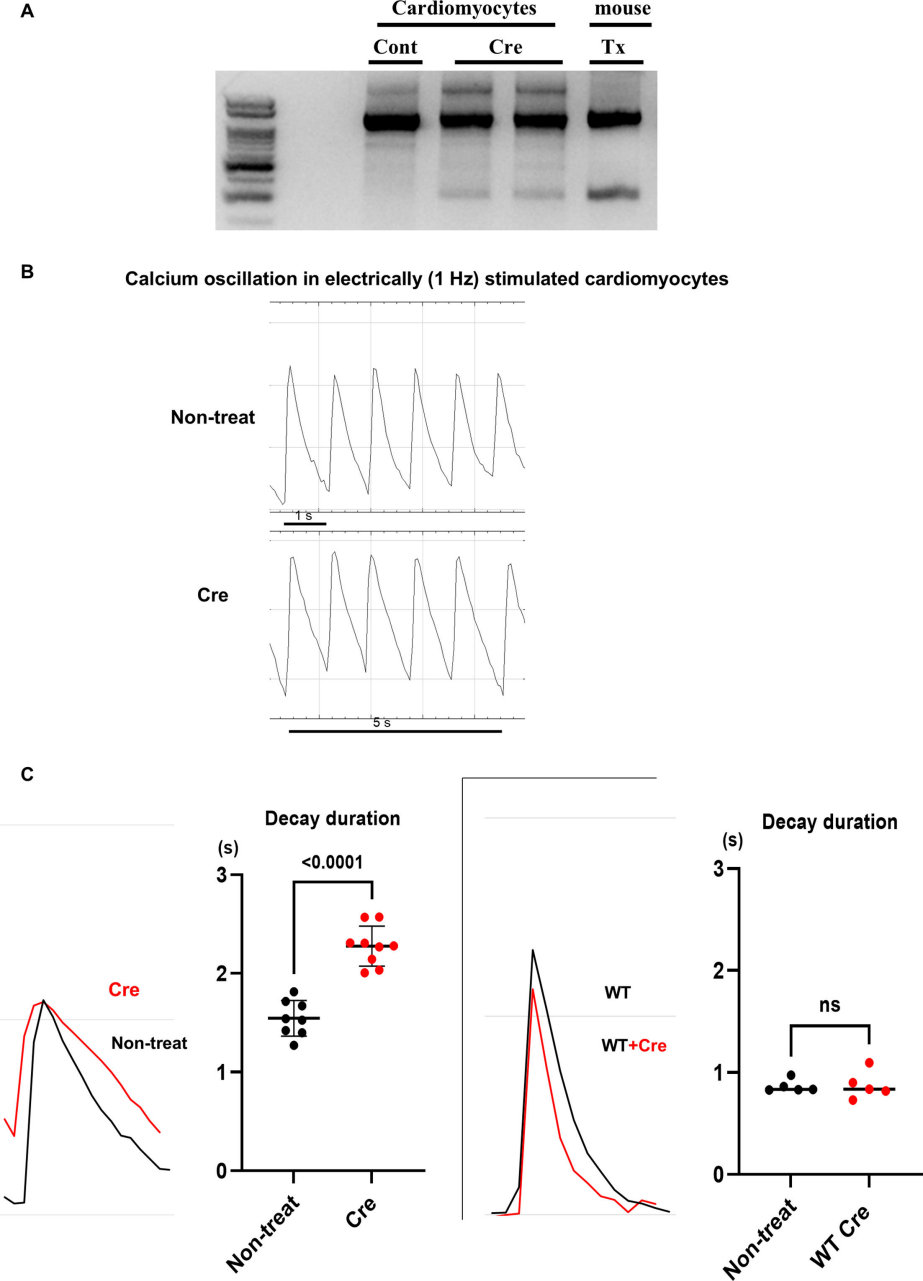
Primary cultured murine fetal cardiomyocytes treated with Cre enzyme influencing calcium spike decay. **A**. Genomic DNA isolated from Cre enzyme-treated primary cultured murine fetal cardiomyocytes showed deletion similar to that from hChAT KO mouse hearts. **B**. Representative calcium oscillation patterns of non-treated (Non-treat) and Cre-treated (Cre) cardiomyocytes, which were electrically stimulated (1 Hz). **C**. Superimposed calcium oscillation curves, set at the peaks, showed a delayed decay pattern of the calcium spike from the peak (tau) in Cre-treated and electrically stimulated cardiomyocytes (Cre) (*P*<0.01). However, WT mice-derived fetal cardiomyocytes were not affected by Cre treatment (WT + Cre), and the spike decay pattern (tau) was comparable with that from non-treated WT cardiomyocytes (Non-treat).

Primary murine fetal cardiomyocytes from *ChAT* ^f/f^ mice were treated with Cre recombinase Gesicle (Takara Bio Inc.) to delete the *ChAT* gene (Figure 9A), and the *ChAT* gene was deleted in the murine heart as shown in Figure 1A. After deletion, when Cre-treated cardiomyocytes were identified by mCherry fluorescence signals, they were loaded with Calbryte^TM^ and electrically stimulated at a fixed frequency (1 Hz) to detect calcium spikes. The representative patterns of calcium spikes in both groups were shown in Figure 9B. Compared with non-treated *ChAT* ^f/f^ mouse cardiomyocytes (Non-treat), Cre-treated cardiomyocytes (Cre) showed that the signals decayed more gradually following the calcium signal peak (Figure 9B and C). The superimposed calcium spike from each cardiomyocyte (Non-treat and Cre), set at each peak, revealed that the signal decay to baseline was delayed in Cre-treated cardiomyocytes compared with non-treated cardiomyocytes (2.28 ± 0.20 s vs.1.55 ± 0.18 s, *n* = 8–9, *P*<0.0001; Figure 9C). However, as shown in the insertion, calcium decay of WT cardiomyocytes without electrical stimulation was comparable between WT cardiomyocytes treated with Cre (WT + Cre) and without Cre (WT) (0.82 ± 0.06 s vs. 0.90 ± 0.11 s, ns, *n* = 6 in Figure 9C), suggesting that Cre Gesicles do not affect calcium signaling, whereas *ChAT* gene deletion does. These results demonstrate that *ChAT* gene deletion in cardiomyocytes influences calcium spikes, especially during the decay phase, and that one of the underlying mechanisms may contribute to mitochondrial dysfunction.

### Down-regulation of the NNCCS causes systemic inflammation

**Figure 10:**
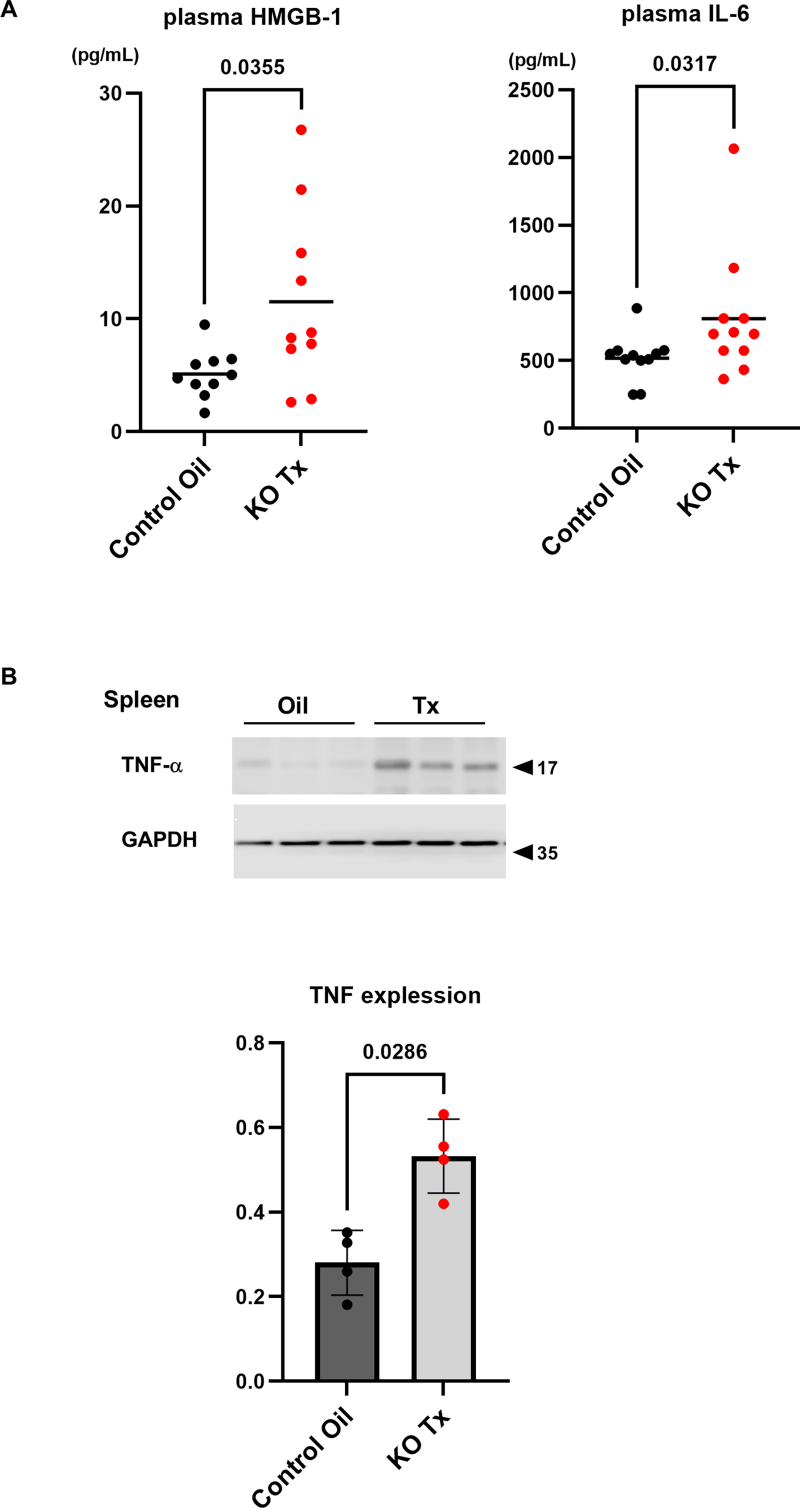
Induction of systemic inflammation in hChAT KO mice despite the heart-specific down-regulation of *ChAT* gene. **A**. Plasma HMGB-1 and IL-6 levels were both significantly up-regulated in hChAT KO mice (*P*<0.05 and *P*<0.05, respectively). **B**. In the spleen, TNF-α protein expression levels were significantly increased in hChAT KO mice (Tx) (*P*<0.05).

The systemic cholinergic system has been known to play an anti-inflammatory role, which has been especially advocated since an epoch study identified an anti-endotoxemia effect of vagus nerve stimulation [20]. Other than the systemic modality modulating vagus nerve functions, the stimulation of the local cholinergic pathway was reported to exert its anti-inflammatory effects [21]. Therefore, to investigate whether down-regulation of the NNCCS triggers inflammation, blood levels of HMGB1 and IL-6 were measured in hChAT KO mice (Figure 10). As expected, plasma levels of HMGB1 were significantly up-regulated in hChAT KO mice (KO Tx), compared with control mice (Control Oil) (11.5 ± 7.9 pg/ml vs. 5.6 ± 2.6 pg/ml, *n* = 10, *P*=0.0355) and those of IL-6 were also significantly elevated in hChAT KO mice (810.4 ± 469.8 pg/ml vs. 517.5 ± 169.9 pg/ml, *n* = 11, *P*=0.0317) (KO Tx). In the spleen, the expression levels of TNF-α protein were also enhanced in hChAT KO mice (Tx), compared with those in control mice (*n* = 4, *P*=0.0286) (Oil). These results suggest that down-regulation of the NNCCS, even in a heart-specific manner, induces systemic inflammation.

### Down-regulation of the NNCCS influencing the BBB function

**Figure 11:**
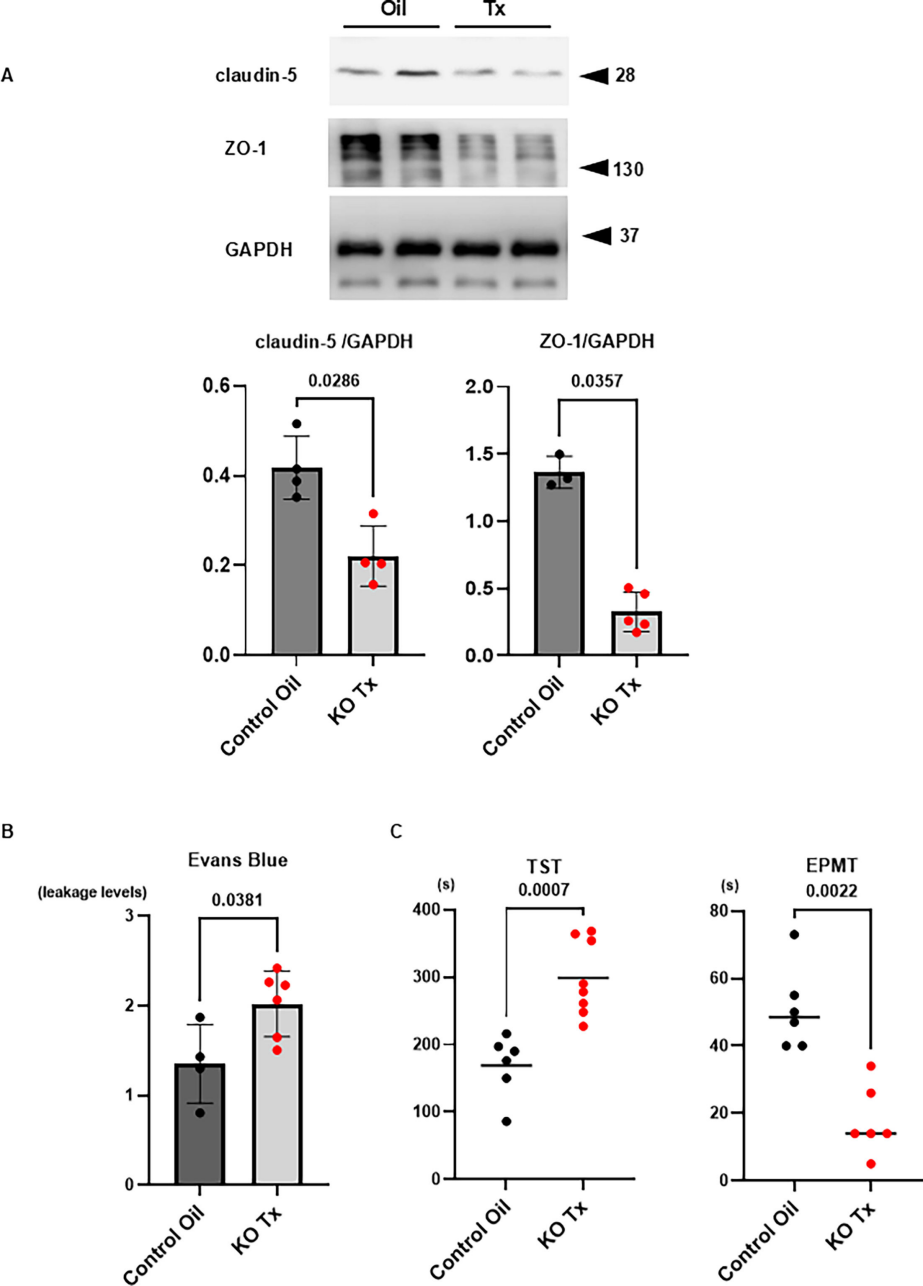
Influences of hChAT KO on the BBB, its function, and depression or anxiety-like phenotypes. **A**. BBB composing proteins, claudin-5 and ZO-1, were down-regulated in ChAT KO mice (KO Tx) (*P*<0.05 each), resulting in significantly more EB leakage in the brain of hChAT KO mice (KO Tx), compared with that in control mice brains (Control Oil) (*P*<0.05). **B**. In hChAT KO mice (KO Tx), the immobilized duration was significantly prolonged (TST), and duration in an open arm of EPM was significantly decreased (EPMT), compared with control mice (Control oil) (*P*<0.01 and *P*<0.01, respectively).

Our previous study demonstrated that NNCCS activation strengthens BBB function and protects the BBB from disruption under pathological conditions [19]. Therefore, the protein expression levels of BBB components in the brain were compared between the control and hChAT KO mice (Figure 11A). The expression levels of claudin-5 (0.221 ± 0.067 vs. 0.418 ± 0.070, *n* = 4, *P*=0.0286) and ZO-1 (0.327 ± 0.146 vs. 1.364 ± 0.120, *n* = 3–5, *P*=0.0357) were significantly attenuated in hChAT KO mouse brains (KO Tx), compared with control mouse brains (Control Oil). Consistently, when hChAT KO mice (KO Tx) were subjected to cold brain injury, a common model to locally disrupt BBB, the leakage of Evans blue dye, which was systemically injected, was significantly aggravated, compared with control mice (Control Oil) (1.95 ± 0.39 vs. 1.35 ± 0.44, *n* = 4–6, *P*=0.0381; Figure 11B). It has been suggested that the down-regulation of NNCCS impairs BBB function.

Additionally, the duration of immobilization was significantly prolonged in hChAT KO mice (KO Tx) subjected to the tail suspension test, a common test for inducing depression-like behavior, compared with control mice (Control Oil) (299 ± 56 s vs. 169 ± 46 s, *n* = 6–10, *P*=0.0007; Figure 11C). In addition, in EPMT, hChAT KO mice preferred the closed arm, and the staying time in an open arm was significantly decreased (17.8 ± 10.4 s vs. 50.8 ± 12.3 s, *n* = 6, *P*=0.0022). Both results suggest that the down-regulation of NNCCS deteriorated the depression and anxiety-like conditions and that this phenomenon was opposite to the central phenotype of NNCCS-up-regulated mice, as reported in our previous studies [11,19].

### Down-regulation of intracellular cholinergic system influencing autophagic responses in AC16 cells

**Figure 12:**
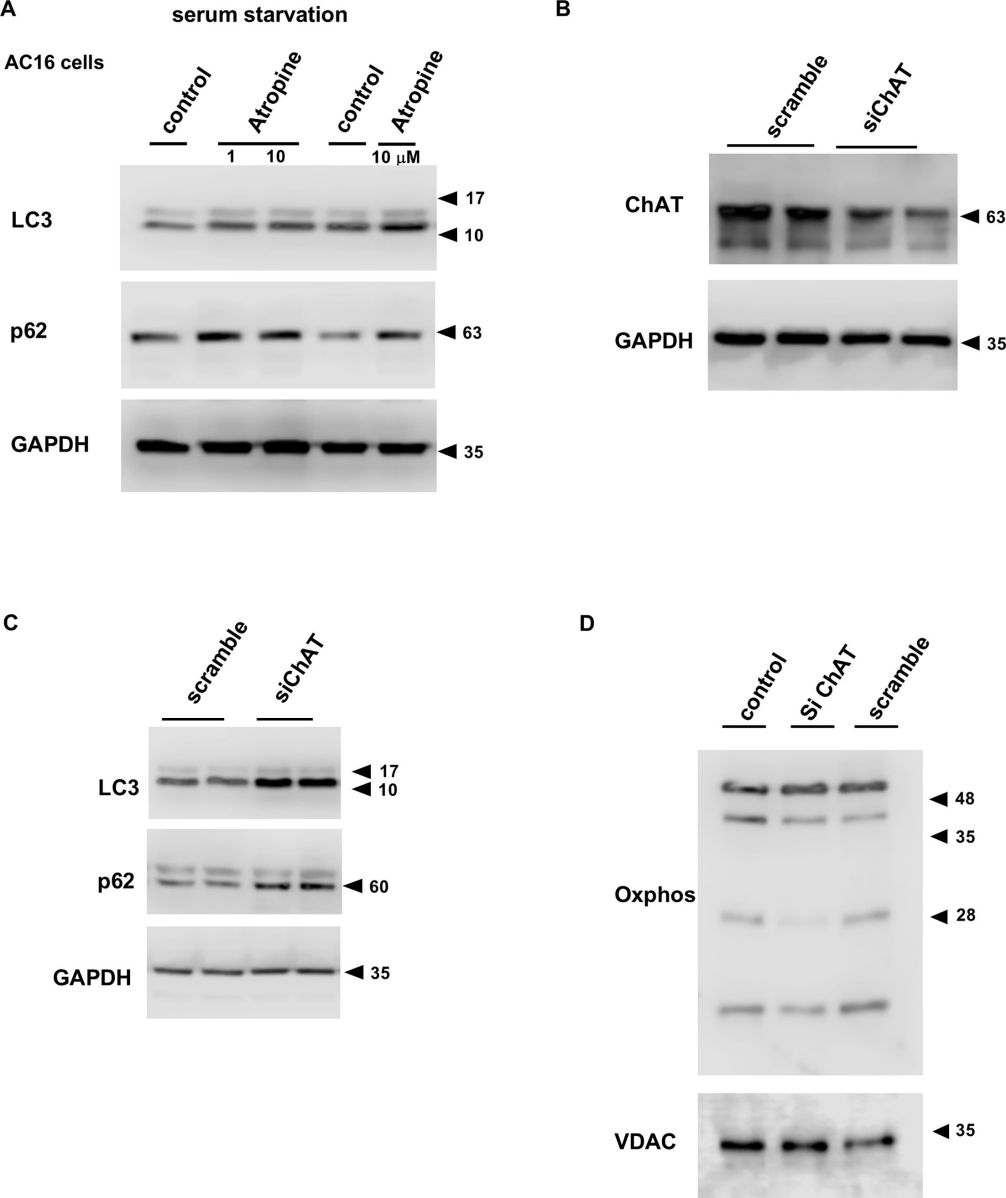
Blockade of the cholinergic signals through muscarinic receptors and down-regulation of the NNCCS up-regulate autophagy. **A**. Serum-starved AC16 cells altered expression of LC3-II and p62 proteins to be increased with the treatment of atropine. **B** and **C**. ChAT mRNA-specific siRNA, siChAT, which decreased ChAT protein expression, alternatively induced LC3-II and p62 protein expression. **D**. siChAT partially decreased OXPHOS protein expression in AC16 cells.

Using a human cardiomyocyte cell line, AC16 cell, the alteration of autophagy was evaluated when cholinergic signals were blocked or *ChAT* gene knockdown was conducted using a commercially validated siRNA for human ChAT mRNA (siChAT). When serum-starved AC cells were treated with atropine, a muscarinic receptor antagonist, autophagy was further induced by increased protein expression of LC3 II and p62 (Figure 12A). siChAT decreased ChAT protein expression in AC16 cells compared with scrambled siRNA (Figure 12B). This suggests that AC16 cells possess a non-neuronal ACh system similar to murine cardiomyocytes. When cells were subjected to siChAT treatment to blunt the non-neuronal ACh system, the protein expression of LC3A II and p62 was up-regulated, suggesting that blunting of the intracellular cholinergic system induces autophagic responses (Figure 12C). These phenomena support our hypothesis that impairment of the NNCCS triggers autophagy. Similar to the mitochondria isolated from the hearts of hChAT KO mice, siChAT-treated AC16 cells showed partially down-regulated expression of OXPHOS proteins in the whole cell lysates, suggesting that *in vitro* findings are compatible with *in vivo* findings thus far demonstrated (Figure 12D).

## Discussion

### Characteristic phenotypes of hChAT KO mice

Utilizing a murine loss-of-function model in the NNCCS, the present study yielded the following novel findings. First, down-regulation of ACh synthesis by cardiomyocytes disrupted cardiac physiological homeostasis. Second, it resulted in the development of cardiac dysfunction or heart failure in mice, as evidenced by cardiac function measurement with a catheter, which revealed systolic and diastolic cardiac dysfunction. Third, the impaired cardiac function caused by blunted NNCCS was associated with morphological alterations in cardiomyocytes, specifically disorganized mitochondria and cardiac muscle fibers, including mitochondrial swelling, segmentation, and deranged muscle fibers. Fourth, failure to sustain mitochondrial integrity morphologically and functionally caused the initiation of autophagic responses. Fifth, mitochondria expressing nAChRs, including α7 nAChR, were involved in calcium entry into the mitochondria in response to nAChR agonists, and mitochondria from the heart with impaired NNCCS attenuated nAChR agonist-mediated calcium accumulation. Sixth, down-regulated NNCCS influenced not only the heart but also extra-cardiac organs, including the brain, and induced systemic inflammation, disruption of BBB function, and depression and anxiety-like phenotypes. Based on these results, the current study is novel in that it not only identified the mitochondria as a critical target of the NNCCS in cardiomyocytes but also demonstrated that the NNCCS is indispensable for sustaining mitochondrial integrity and for regulating the BBB and higher brain functions. Therefore, loss of function leads to cardiac dysfunction associated with disturbed mitochondrial functions, leading to poor energy metabolism, enhanced exposure to ROS, and failure to properly handle calcium in the heart and disturbed function in the brain.

### The cardiac cholinergic system composed of the vagus nerve and NNCCS

The non-neuronal ACh system in the heart was initially reported by a research group that revealed ChAT-positive immunoreactivity in frog hearts [22]. However, the authors did not report on the physiological or pathological significance of this system in cardiomyocytes. More than 10 years later, the physiological relevance of the non-neuronal ACh system in the heart was reported, indicating that the system negatively regulates energy metabolism in cardiomyocytes and plays a role in protecting the heart from ischemia or hypoxic insults [10]. Later, we referred to this as the NNCCS [11–13, 19]. This study [10] was almost simultaneously followed by that of Rana OR, et al., who reported that this system is down-regulated with age [6]. Thereafter, the other researchers, Rocha-Resende C and Roy A et al., also revealed the physiological and pathophysiological roles of NNCCS using acetylcholinesterase inhibitors and genetically modified mice [7,8,14]. Based on these numerous studies by independent researchers worldwide, it has been recognized that the system—that is, the NNCCS or non-neuronal ACh in the heart—exists there, and the heart is regulated by two systems: the PNS and NNCCS.

### Impaired cardiac dysfunction with higher mortality in conditional hChAT KO mice without angiotensin II loading

Regarding genetically modified mice in the NNCCS, especially loss-of-function models, several types of models have been reported, for example, heart-specific conditional *ChAT* gene or *VAChT* gene-deleted mice using Myh6-Cre [8,14], and heart-specific ChAT mRNA knockdown mice [16]. Roy et al. reported the significance of the NNCCS using conditional ChAT and VAChT KO mice when they were finally subjected to angiotensin II treatment [8,14] because both heart-specific conditional KO mice showed comparable cardiac function to non-KO mice without angiotensin II stress. However, this function was impaired following angiotensin II loading, and both models were further associated with aggravated cardiac remodeling. In contrast, heart-specific VAChT transgenic mice were resistant to angiotensin II-mediated stress, and cardiac remodeling was significantly attenuated, which was consistent with the phenotypes of heart-specific ChAT transgenic (hChAT tg) mice [13], strongly supporting the hypothesis that the activation of NNCCS plays a protective role in hypoxic or ischemic insults and attenuates cardiac remodeling [7, 8, 12–14]. These findings indicate that the NNCCS plays a role in attenuating cardiac remodeling and dysfunction under pathological conditions.

However, compared with those studies [8,14], the current study using conditional hChAT KO mice presented novel and distinctive findings. First, hChAT KO mice exhibited more drastic and severe cardiac dysfunction during the early phase after Tx injection; some of these mice showed a typical heart failure-like phenotype and ultimately died. In such severely compromised conditions, the cardiac function of KO mice could not be easily assessed through a catheter-based technique due to unstable hemodynamics. Therefore, cardiac function was evaluated using mice that survived during the chronic phase. This reminds us that compared with dead KO mice, live KO mice probably possess less compromised cardiac function. Furthermore, following the death of KO mice due to pump failure in the early phase, it was observed that some of the surviving mice suddenly died. Notably, KO mice rarely exhibited arrhythmias, such as conduction blocks (data not shown). The recent report indicates that this system is involved in regulating electrical conduction in the heart [23], suggesting increased arrhythmogenicity in the hearts of hChAT KO mice. This arrhythmogenicity, together with dysfunctional mitochondria by reduced membrane potential and increased cytochrome c and ROS release, may contribute to the mortality of hChAT KO mice. In contrast to previous studies [8,14], this study revealed that hChAT KO mice suffered from cardiac dysfunction in the absence of cardiac stress, exemplified by the load of angiotensin II. The reason why the severity of hChAT KO mice differed from that of other KO mice [14] remains unknown. Possible explanations could be that even in so-called heart-specific ChAT KO mice, flox-flanking regions in the *ChAT* gene might differ from each other, the overexpression levels of Cre in the heart might be different, or our hChAT KO mice were developed from the initial step under the C57BL/6 J mouse genetic background and consequently did not need a backcross. In addition, our previous study demonstrated that heart-specific ChAT knockdown (hChAT KD) mice with ChAT-specific miRNA overexpression, likewise, suffered from impaired cardiac dysfunction without cardiac stress, such as with angiotensin II, in adulthood [16]. Although the onset time of cardiac dysfunction differed between hChAT KO mice and hChAT KD mice, the shared and common evidence of cardiac dysfunction without coadministration of angiotensin II contrasts with that of other studies [8,14]. These results indicate the critical role of the NNCCS in sustaining cardiac function even in a physiologically steady state.

### The NNCCS influencing mitochondrial functions

Experiments evaluating protein expression levels in the heart revealed that the levels of GLUT-4 and the p-Akt/Akt ratio were both down-regulated in hChAT KO mice, suggesting that hChAT KO cardiomyocytes did not efficiently utilize the energy substrate glucose. These results were also consistent with those of our previous studies using hChAT KD mice, in which the expression levels of HIF-1α, GLUT-4, and the p-Akt/Akt ratio were all significantly decreased [16]; however, the expression of these markers was reciprocally up-regulated in the hearts of hChAT tg mice [13]. The result that the alteration of the NNCCS influences cardiac energy metabolism prompted us to speculate whether the NNCCS specifically regulates mitochondrial functions and morphological structures. Intriguingly, evidence that NNCCS targets mitochondria and influences their function was confirmed by a morphological experiment in cardiomyocytes, as shown in Figure 2B. Electron microscopy revealed more disrupted and smaller-segmented mitochondria, accompanied by a swollen appearance and destructive cristae in hChAT KO hearts.

Furthermore, the mitochondrial membrane potential of the heart is influenced by the down-regulation of the NNCCS. As shown in Figure 5, hChAT KO mice-derived mitochondria tended to lose those with higher potential than control mice-derived mitochondria. This suggests that disrupted intracellular cholinergic signals result in down-regulated mitochondrial membrane potential; however, the precise mechanisms by which NNCCS-derived ACh regulates and sustains this potential remain obscure. Some previous studies have revealed that ACh-treated cells sustained the intact mitochondrial membrane potential even in chemically hypoxic conditions, which generally decrease the potential without ACh [24,25]; consequently, these findings also support and consolidate our current speculation and hypothesis.

The copy number of mitochondrial DNA from the heart was significantly reduced, and mitochondria labeling with MitoTracker^®^ revealed the reduction in the number. Functionally, cardiac ATP levels were decreased, accompanied by decreased mitochondrial proteins involved in the electron transport chain, as shown in Figure 3. In addition, cytochrome c release was enhanced in the cytosolic fraction, and the range of mitochondrial membrane potential shifted toward lower levels in hChAT KO hearts. Consequently, the heart was more exposed to ROS, as evidenced by the elevated nitrosylation levels and attenuated reducing states of the heart tissue. These phenotypes of malfunctioning cardiomyocytes with ROS burden in hChAT KO mice were also observed and shared by hChAT KD mouse hearts [16], as well as the loss-of-function model in a non-neuronal ACh system *in vitro* reported in our previous study [12], which revealed that the *in vitro* loss-of-function exposed cardiomyocytes to more ROS and induced apoptosis, particularly when they were stimulated with norepinephrine [12]. Collectively, these results indicate that the NNCCS is crucial for functional and physiological cardiac stability, even under non-stressful conditions and in pathological conditions, because the NNCCS influences mitochondrial function.

### Inactivation of the NNCCS inducing autophagy targeting mitochondria

The finding that NNCCS targets mitochondria is also supported by the induction of autophagy in the heart, as demonstrated in Figure 4. In an *in vivo* situation, hChAT KO mouse hearts showed increased p62 accumulation and LC3II expression, and a cytosolic fraction of the hChAT KO heart (Tx) decreased the expression levels of Parkin and PINK. However, these protein levels reciprocally increased in the mitochondrial fraction in hChAT KO hearts (Tx), indicating that autophagy is induced in response to the destruction of mitochondria, as clearly shown in Figure 2B. In addition, mitochondria from hChAT KO mouse hearts showed a reduction in mitochondria-related electron transport system proteins, as shown in Figure 3. Similarly, as shown in Figure 12, when AC cells were treated with atropine, LC3 expression levels were further altered, and p62 protein expression levels were up-regulated. Moreover, ChAT knockdown with a ChAT mRNA-specific siRNA induced autophagic responses and partially down-regulated OXPHOS expression (Figure 12A, B and D), as shown in hChAT KO hearts. These results suggest that the blockade of the cholinergic signaling pathway, whichever extra- or intracellular pathway, may trigger a signal that accelerates the autophagic response. Therefore, the NNCCS is suggested to be crucial for sustaining mitochondrial integrity and suppressing the breakdown of mitochondria-related proteins.

### Mitochondria involved in regulating cytosolic calcium through calcium entry mediated by nAChR stimulation

Another important and novel finding in this study is that using a pharmacological approach, isolated mitochondria from WT mouse hearts were found to modulate calcium entry through nAChRs, which are located on the mitochondrial membrane (Figure 7). Western blot analysis confirmed that α7 nAChR signals were found predominantly in the mitochondrial fraction, whereas a few signals were detected in the cytoplasmic fraction. Moreover, an immunocytochemical study demonstrated the co-localization of MitoTracker Orange signals and α7 nAChR signals. To the best of our knowledge, this result is novel for cardiomyocytes. Our finding that mitochondria of murine cardiomyocytes express α7 nAChRs is supported by previous studies by Skok M et al., who reported that signals of α7 nAChR immunoreactivity were observed predominantly in the mitochondria of human astrocytes and B cells [17,26]. α7 nAChR is composed of homopentameric subunits that play a role in conducting calcium influx in response to receptor stimulation. It is an abundant representative nAChR, along with α4/β2 nAChRs, in the brain [27].

Mitochondria isolated from WT hearts responded to ACh or several α7 nAChR agonists, GST-21 or PNU289785, to increase mitochondrial calcium levels. Their responses were blunted by a non-specific nAChR antagonist, mecamylamine, or α7 nAChR-specific antagonist, methyllycaconitine, respectively. Since calcium elevation levels and the antagonizing effects were influenced by variations in individual mitochondrial sampling procedures, it was difficult to differentiate pharmacologically which nAChRs were mainly involved in mitochondrial calcium uptake, including α7 nAChR. However, according to our results, not only α7 nAChR but also other nAChRs may be responsible for the uptake, although little information is available at present on how abundantly each nAChR exists in mitochondria. In contrast, in the hearts of KO mice with malfunctioning and damaged mitochondria, the deranged and impaired functions of nAChRs resulted in failure to precisely handle calcium in the mitochondria to regulate calcium levels in the cytosol. Consequently, it is at least indicated that some nAChRs, including α7 nAChR in mitochondria, are responsible for calcium uptake to regulate a cytosolic calcium level in cardiomyocytes. These results suggest that ACh synthesized by cardiomyocytes influences mitochondrial calcium uptake through nAChRs, resulting in the buffering of cytosolic calcium.

Furthermore, in mitochondria, an MCU is located on the outer membrane and is involved in transporting calcium from the cytosol to the mitochondria [28,29]. As a player in the regulation of cytosolic calcium levels in cardiomyocytes, SERCA2 is a major regulator of calcium uptake into the endoplasmic reticulum. However, both the MCU and α7 nAChRs identified in this study are considered responsible for calcium handling because the loss of function in the MCU causes impaired calcium handling by mitochondria [30]. A physiological increase in mitochondrial calcium content activates energy metabolism in the heart by accelerating TCA cycle flux to produce more ATP, contributing to the up-regulated activities of Complexes I, III, and IV [30]. In contrast, further increased calcium levels in the mitochondria, that is, mitochondrial calcium overload, are alternatively associated with impaired mitochondrial respiration and activation of mitochondrial permeability transition pores [31]. The function of the MCU is tightly regulated through its compensation in the heart. This is clarified by the fact that the heart-specific MCU knockout mice do not exhibit alterations in mitochondrial calcium levels or cardiac basal functions under non-stressful and basic conditions [30]. In contrast, under stressed conditions, such as catecholamine load or physical activity, MCU KO mice showed impaired responses in terms of cardiac contraction and relaxation [32,33]. These studies suggest that a properly regulated MCU is required to play a role in tightly tuning calcium levels and energy metabolism to maintain cardiac functions. From the viewpoint of calcium handling by mitochondria, the protein levels of MCU and α7 nAChR decreased following Tx injection (hChAT KO mice), partly reflecting the reduction in intact mitochondria, as evidenced by western blot analysis and electron microscope observation. Moreover, mitochondria isolated from hChAT KO mice showed decreased, but not increased, calcium signals in response to ACh (Figure 8), in contrast with the mitochondrial responses from WT mouse hearts. According to either reduced MCU or failed nAChR responses in the mitochondria, the failure to accumulate adequate calcium in the mitochondria via these two pathways may contribute to energy metabolic dysfunction in the heart. Whether the MCU is regulated by nAChR responses or interacts with α7 nAChR remains elucidated. However, it is speculated that the functions of the mitochondria-related calcium-modulating proteins MCU and α7 nAChR may be impaired and contribute to cardiac dysfunction in hChAT KO mice.

As already mentioned, Skok M et al. reported the localization of α7 nAChR on the outer membrane of mitochondria of extra-cardiac cells [17,26]; however, studies other than the current one have never reported the existence of nAChR on mitochondria in cardiomyocytes. Therefore, this study provides intriguing evidence that can support our understanding of how the loss of function in the NNCCS is linked to malfunctioning cardiomyocytes through nAChRs in the mitochondria.

Of note, compared with our investigation, the previous studies by Skok M. et al. reported calcium efflux from the mitochondria of isolated hepatocytes stimulated with ACh or α7 nAChR agonist, suggesting that calcium was partially released from the mitochondria in response to nAChRs [17], in contrast to our current results. Using the same calcium assay buffer used to suspend mitochondria, we also observed calcium signals in supernatants after the mitochondria were suspended in a fresh buffer with ACh, suggesting calcium efflux from the mitochondria. However, by simultaneously further increasing the amount of calcium imported into the mitochondria, the accumulation levels were elevated compared with non-stimulated mitochondria. Consequently, this phenomenon does not exclude the possibility of the partial export of calcium from the mitochondria.

### hChAT KO mouse hearts with diastolic and systolic dysfunctions

The present study revealed that after Tx injection, some KO mice died in the early phase, probably due to heart failure, before the cardiac function study was conducted. Therefore, only the mice that were left alive were subjected to the cardiac function study, suggesting that the results pertaining to cardiac function might have been underestimated. Nevertheless, the hearts of hChAT KO mice showed not only systolic dysfunction but also diastolic dysfunction because the levels of -dP/dt and Tau were significantly altered in the hearts of hChAT KO mice, indicating that diastolic function was also impaired. This was supported by the results of primary cardiomyocytes obtained from fetal mice possessing a homologous flox-flanked *ChAT* gene. In Cre-transfected cardiomyocytes, that is, ChAT KO cardiomyocytes, specified by mCherry fluorescence, calcium oscillation was evaluated with electrical stimulation at a fixed frequency (1 Hz), and compared with non-transfected cardiomyocytes or Cre-transfected WT cardiomyocytes, both of which were stimulated at 1 Hz. The decay of calcium spikes in ChAT KO cardiomyocytes was significantly slower than that in non-KO cardiomyocytes, suggesting that cytosolic calcium is not efficiently and tightly regulated following the blunting of the NNCCS, as shown in Figure 9. Taken together with the *in vivo* evidence of impaired ACh-induced mitochondrial calcium uptake and loss of MCU, it is speculated that mitochondrial dysfunction with morphological alterations caused by blunting of the NNCCS may impair calcium handling and elevate cytosolic calcium levels, that is, calcium overload in the cytosol, leading to impaired diastolic function.

### The link between the NNCCS and the BBB

This study revealed another phenotype regarding the attenuated expression of BBB-composing proteins induced by NNCCS down-regulation, as shown in Figure 11. In KO mice, the expression levels of claudin-5 and ZO-1 were attenuated, suggesting BBB malfunction [34,35]. A BBB function is often evaluated using a cold brain injury model, in which the BBB is tentatively disrupted with the local leakage of systemically administered Evans blue, followed by the gradual recovery [36,37]. Compared with control mice (Oil), hChAT KO mice showed significantly enhanced Evans blue leakage (Tx). This phenotype is in contrast to the brain phenotype of heart-specific ChAT (hChAT) tg mice [19], which showed enhanced BBB function via increased expression of claudin-5 protein and resilience against pathological neuroinflammatory insults [19]. Consequently, they possess anti-inflammatory potential in both the heart and brain [13,19], which is dependent on the afferent vagus nerve pathway stimulated by increased nitric oxide from the heart [11]. These phenotypes of hChAT tg mice are considered to possess two sides of the same coin as those of hChAT KO or hChAT KD mice [16].

hChAT KO mice demonstrated systemic inflammatory reactions represented by elevated blood levels of HMGB1 and IL-6, associated with elevated TNF-α expression in the spleen, contrasting to the anti-inflammatory reactions by hChAT tg mice [13,19]. Such systemic inflammation itself may be involved in the down-regulation of the BBB function because pro-inflammatory cytokines are reported to blunt the expression of BBB components [38]; in contrast, the cholinergic system itself, whether local or systemic, conducts anti-inflammatory actions [3,21,39]. Hence, in hChAT KO mice, impaired heart-derived signaling of the NNCCS may dampen both anti-inflammatory effects and sustaining of the BBB integrity. Consequently, it is strongly suggested that the NNCCS regulates the BBB.

Based on these characteristic phenotypes of hChAT KO mouse brains, that is, loosened and disrupted BBB, it was speculated that higher brain function may be affected [16,19]. Compatibly, hChAT KO mice showed a typical depression-like phenotype, which was evidenced by a significantly increased duration of immobility in the tail suspension test, and an anxiety-like phenotype in EPMT [16]. This is also in contrast to the anti-depression and anti-anxiety-like behavior of hChAT tg [11]. The evidence of a down-regulated NNCCS, leading to the impairment of BBB function and the induction of inflammatory responses, also strengthens our hypothesis that the NNCCS positively regulates the functions of the BBB and the higher brain.

However, the precise pathways by which the NNCCS regulates the BBB function remain to be fully elucidated in this study. Moreover, it remains unclear whether and how the ascending vagus nerve, terminating at the solitary tract nucleus, alternatively propagates its cholinergic or adrenergic signals specifically to the cerebral vasculatures in the whole brain. Several reports revealed that β-adrenergic receptor agonists or catecholamines prevented the tight junction from decreasing functions [19,40,41]. Notably, vagus nerve stimulation has been reported to influence BBB and brain functions [39,42]. In addition, cholinergic stimulant-treated endothelial cells were reported to elevate protein expression of BBB components, including claudin-5 [43,44]. Based on these, it is speculated that the vagus nerve influences BBB functions, probably indirectly, through both the cholinergic and adrenergic pathways. These phenomena are compatible with our current results and strengthen our hypothesis because the central phenotypes of hChAT tg mice overlap extensively with those of vagus nerve-stimulated mice [11,19]. Collectively, both striking but conflicting phenotypes from the brains of hChAT KO mice and hChAT tg mice can shed light on the relationship between the NNCCS and the BBB function, in addition to the influence of the NNCCS on the cardiac function.

The clinical link between depression and heart failure or cardiac diseases in patients has been addressed, with major depressive disorder identified as a risk factor for cardiac events [45,46]. Consequently, the current study may offer insights into the underlying mechanisms of this connection, particularly in relation to NNCCS activity.

### Clinical implications

NNCCS, the system of cardiomyocytes that synthesizes ACh, i.e., non-neuronal ACh from the heart, has been gaining attention in the cardiovascular research field. One reason for this interest is its cardioprotective roles, as evidenced by several transgenic mouse models [13,14]. Conversely, studies have shown that impairing this system can lead to impaired cardiac function and dysregulated autonomic control, highlighting its significance [14,16]. These findings underscore the crucial role of the NNCCS in maintaining cardiac homeostasis and prompt consideration of its clinical application.

Despite its importance, there are few studies that have investigated the relationship between NNCCS and the pathogenesis of cardiac diseases in clinical settings. Our previous research demonstrated that NNCCS is down-regulated during the progression of diabetic hearts in both mice and humans [47]. To the best of our knowledge, this is the first report showing that the down-regulation of NNCCS in human hearts under diabetic conditions. In contrast, activating NNCCS in a murine diabetic model helped sustain cardiac function, bringing it to levels comparable with those of non-diabetic hearts during disease progression [47]. Therefore, activating NNCCS may help mitigate further deterioration of cardiac function in pathological conditions.

Additionally, our earlier study found that donepezil, an anti-Alzheimer’s disease drug, enhances NNCCS activity and increases cardiac ACh levels, suggesting it may act as an inducer or activator of NNCCS [10]. Epidemiological studies have also shown that patients with Alzheimer’s disease, who were prescribed donepezil, experienced significantly fewer cardiovascular events and had better survival rates compared with those who were not treated with the drug [48]. Moreover, patients receiving donepezil were less likely to suffer from acute coronary syndrome, and this effect was observed in a dose-dependent manner [49]. These findings support the notion that pharmacologically activating NNCCS may offer protection for the heart against cardiac diseases. Beyond donepezil, a limited number of studies have reported another potential inducer of NNCCS using murine models [50,51]. Therefore, focusing on NNCCS and its inducer may provide new therapeutic options in clinical practice. It is anticipated that in the near future, the development of a drug specifically designed to induce NNCCS will become a reality.

## Conclusion

In this study, the NNCCS played a critical role in modulating mitochondrial functions, partly through calcium handling. With down-regulated NNCCS, the mitochondria-rich organ, the heart, is more affected, leading to the loss of intact mitochondrial architectures and functions, impaired cardiac energy metabolism, and the induction of autophagy. However, such compensatory responses cannot compensate for cardiac function to an intact level, indicating that the NNCCS is an intrinsic and fundamental machinery that sustains cardiac function by targeting mitochondria. The NNCCS further plays a role in modulating functions of the BBB and brain and is essential for sustaining the physiological activities.

**Clinical Perspectives**

- Non-neuronal acetylcholine (ACh) synthesis system is equipped with cardiomyocytes to produce ACh, which is an essential factor to maintain cardiac physiological functions; however, it remains to be elucidated how cardiomyocyte-derived ACh plays a specific role in cardiomyocytes to regulate the functions.
- The loss of function of this system caused cardiac dysfunction, which was associated with morphological and functional disturbance of mitochondria, enhancement of autophagy, and induction of systemic inflammation, leading to blood-brain barrier dysfunction and impaired higher brain function.
- Impairment of the non-neuronal ACh synthesis system could be a potential cause of cardiac dysfunction; in contrast, up-regulation of this system may be a therapeutic option against heart failure.

## Supplementary material

online supplementary material 1

online supplementary material 2

online supplementary material 3

## Data Availability

All relevant data that support the findings of this study are available from the corresponding author upon reasonable request.

## Open Access

This article has been published open access under our Subscribe to Open programme, made possible through the support of our subscribing institutions, learn more here: https://portlandpress.com/pages/open_access_options_and_prices#conditional

## Abbreviations

ACh: acetylcholine
ChAT: choline acetyltransferase
MCU: mitochondrial calcium uniporter
NNCCS: non-neuronal cardiac cholinergic system
