# Supplementary material for: Impaired cardiac non-neuronal acetylcholine synthesis triggers mitochondrial dysfunction with the loss of nicotinic receptor-mediated calcium handling, causing the failing heart
Source: Clin Sci (Lond). 2025 Nov 25;139(22):1543–70. doi: 10.1042/CS20257026 (PMC12751086; doi:10.1042/CS20257026)
Supplement: online supplementary material 1 [file CS-139-22-CS20257026-s001.pdf]

Supplemented Figure A

Time course alterations of plasma HMGB1, and cardiac BNP mRNA, ANP and GLUT4, together with DRP1 and Mitofusin

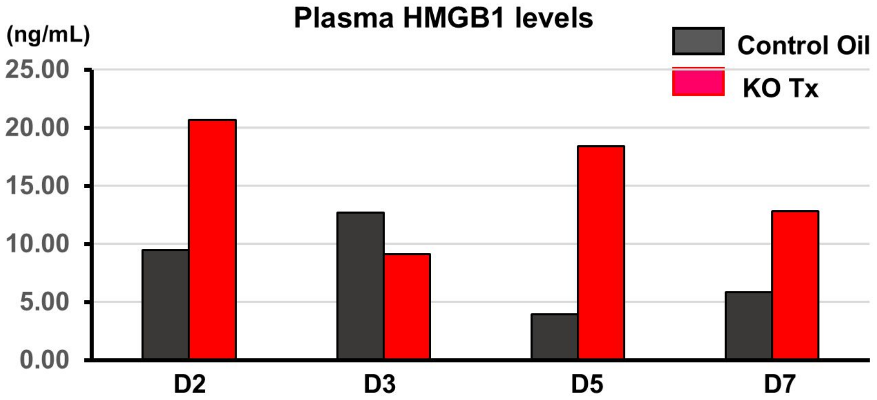

Plasma levels of HMGB1 in hChAT KO mice (KO Tx) show an increased trend first on day 2, compared to those in Control mice (Control Oil), followed by almost sustaining the trend (n=1-2 in each time point).

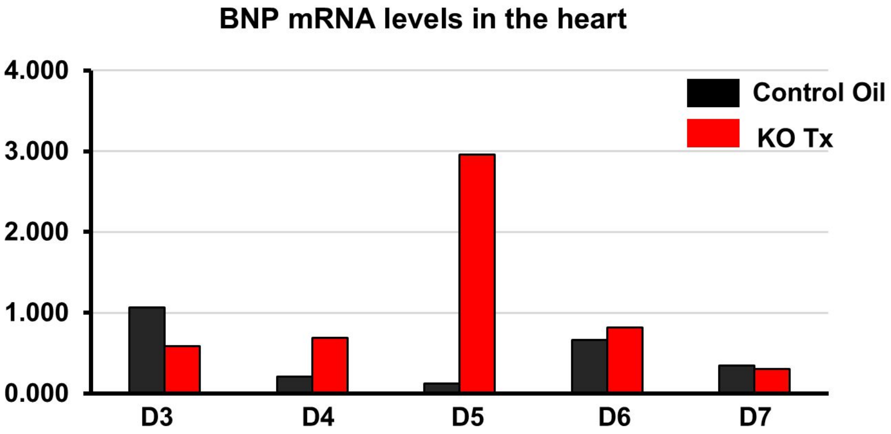

The increase in BNP mRNA in KO Tx begins from day 4; however, it occurs later than that of plasma HMGB1 (n=1-2 in each time point).

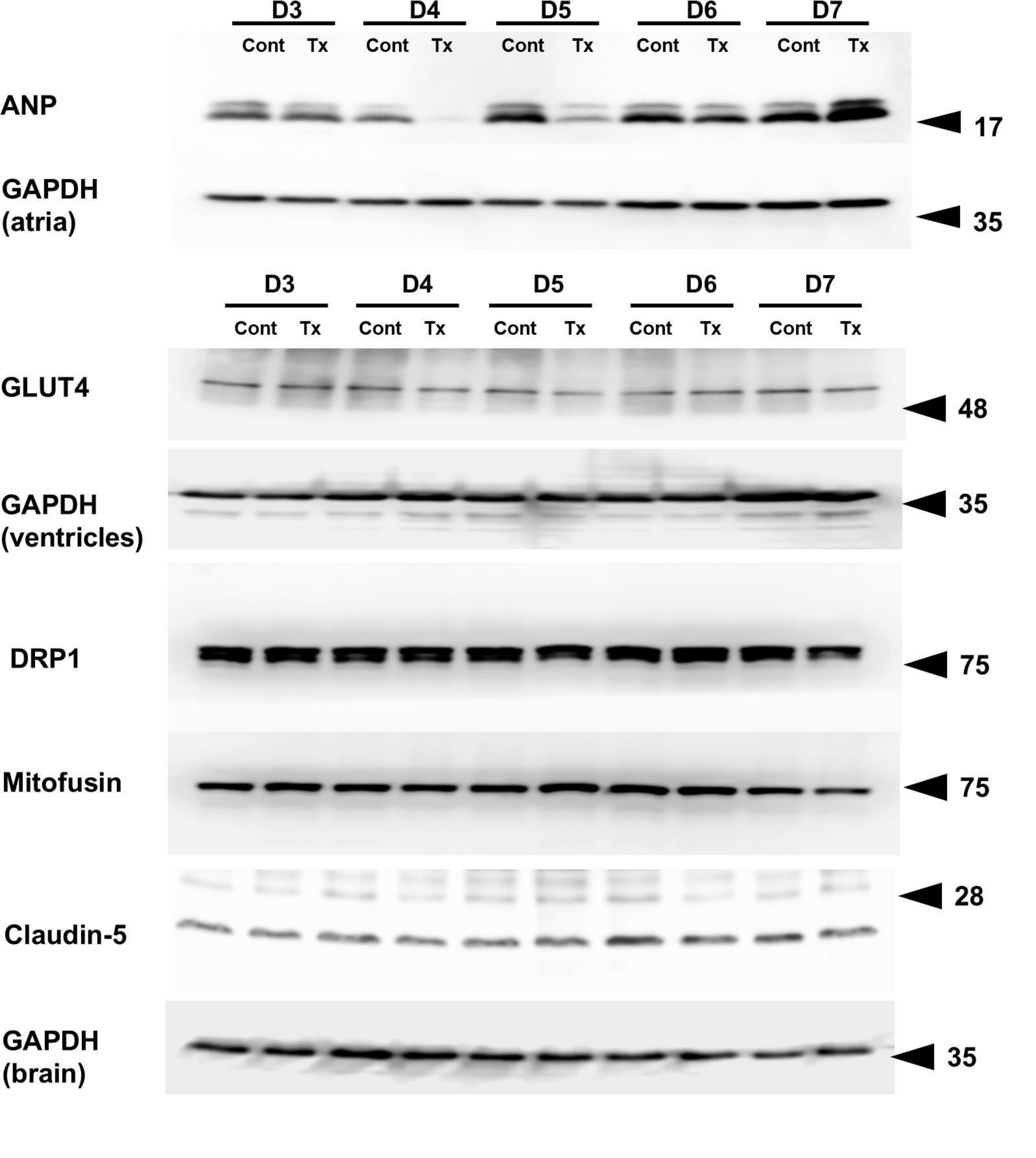

The downregulation of GLUT4 protein levels begins on day 4 in hChAT KO, and subsequently, on day 7, ANP levels are upregulated. However, DRP1 and Mitofusin-2 levels are not altered.

Supplemented Figure B

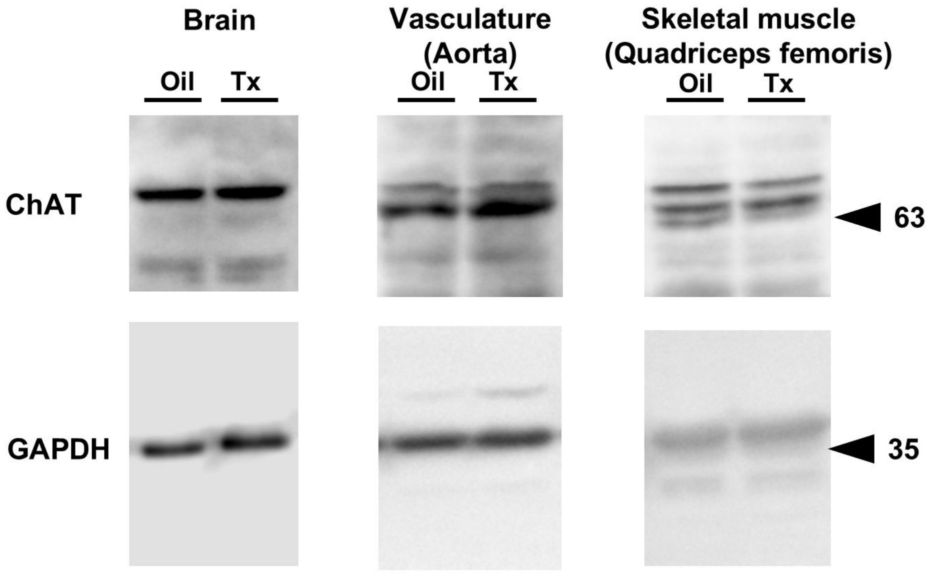

ChAT protein expression levels are comparable between Oil and Tx in the brain, vasculature, and skeletal muscle.
